# Mapping prenatal predictors and neurobehavioral outcomes of an epigenetic marker of neonatal inflammation – A longitudinal population-based study

**DOI:** 10.1016/j.bbi.2024.08.053

**Published:** 2024-08-29

**Authors:** Anna Suleri, Nicole Creasey, Esther Walton, Ryan Muetzel, Janine F. Felix, Liesbeth Duijts, Veerle Bergink, Charlotte A.M. Cecil

**Affiliations:** a Department of Child and Adolescent Psychiatry/Psychology, Erasmus MC, University Medical Center Rotterdam, Rotterdam, the Netherlands; b The Generation R Study Group, Erasmus MC, University Medical Center Rotterdam, Rotterdam, the Netherlands; c Department of Clinical, Educational & Health Psychology, Division of Psychology & Language Sciences, Faculty of Brain Sciences, University College London, London, UK; d Department of Psychology, University of Bath, Bath, UK; e Department of Radiology and Nuclear Medicine, Erasmus MC, University Medical Center Rotterdam, Rotterdam, the Netherlands; f Department of Pediatrics, Erasmus MC, University Medical Center Rotterdam, Rotterdam, the Netherlands; g Department of Pediatrics, Division of Respiratory Medicine and Allergology, Erasmus MC, University Medical Center Rotterdam, Rotterdam, the Netherlands; h Department of Neonatal and Pediatric Intensive Care, Division of Neonatology, Erasmus MC, University Medical Center Rotterdam, Rotterdam, the Netherlands; i Department of Psychiatry, Icahn School of Medicine at Mount Sinai, New York, NY, USA; j Department of Psychiatry, Erasmus MC, University Medical Center Rotterdam, Rotterdam, the Netherlands; k Department of Epidemiology, Erasmus MC, University Medical Center Rotterdam, Rotterdam, the Netherlands; l Department of Biomedical Data Sciences, Molecular Epidemiology, Leiden University Medical Center, Leiden, the Netherlands

**Keywords:** Epigenetics, DNA methylation, Neonatal inflammation, Neonatal infection, Cord blood, Neurodevelopment

## Abstract

**Background:**

DNA methylation levels at specific sites can be used to proxy C-reactive protein (CRP) levels, providing a potentially more stable and accurate indicator of sustained inflammation and associated health risk. However, its use has been primarily limited to adults or preterm infants, and little is known about determinants for – or offspring outcomes of – elevated levels of this epigenetic proxy in cord blood. The aim of this study was to comprehensively map prenatal predictors and long-term neurobehavioral outcomes of neonatal inflammation, as assessed with an epigenetic proxy of inflammation in cord blood, in the general pediatric population.

**Methods:**

Our study was embedded in the prospective population-based Generation R Study (n = 2,394). We created a methylation profile score of CRP (MPS-CRP) in cord blood as a marker of neonatal inflammation and validated it against serum CRP levels in mothers during pregnancy, as well as offspring at birth and in childhood. We then examined (i) which factors (previously associated with sustained inflammation) explain variability in MPS-CRP at birth, including a wide range of prenatal lifestyle and clinical conditions, pro-inflammatory exposures, as well as child genetic liability to elevated CRP levels; and (ii) whether MPS-CRP at birth associates with child neurobehavioral outcomes, including global structural MRI and DTI measures (child mean age 10 and 14 years) as well as psychiatric symptoms over time (Child Behavioral Checklist, at mean age 1.5, 3, 6, 10 and 14 years).

**Results:**

MPS-CRP at birth was validated with serum CRP in cord blood (cut-off > 1 mg/L) (AUC = 0.72). Prenatal lifestyle pro-inflammatory factors explained a small part (i.e., < 5%) of the variance in the MPS-CRP at birth. No other prenatal predictor or the polygenic score of CRP in the child explained significant variance in the MPS-CRP at birth. The MPS-CRP at birth prospectively associated with a reduction in global fractional anisotropy over time on mainly a nominal threshold (β = −0.014, SE = 0.007, p = 0.032), as well as showing nominal associations with structural differences (amygdala [(β = 0.016, SE = 0.006, p = 0.010], cerebellum [(β = −0.007, SE = 0.003, p = 0.036]). However, no associations with child psychiatric symptoms were observed.

**Conclusion:**

Prenatal exposure to lifestyle-related pro-inflammatory factors was the only prenatal predictor that accounted for some of the individual variability in MPS-CRP levels at birth. Further, while the MPS-CRP prospectively associated with white matter alterations over time, no associations were observed at the behavioral level. Thus, the relevance and potential utility of using epigenetic data as a marker of neonatal inflammation in the general population remain unclear. In the future, the use of epigenetic proxies for a wider range of immune markers may lend further insights into the relationship between neonatal inflammation and adverse neuro development within the general pediatric population

## 1. Introduction

Inflammation is increasingly recognized as an important player in both the development and progression of psychiatric disorders, such as depression and attention-deficit/hyperactivity disorder (ADHD) (Han et al., 2021; Thylur and Goldsmith, 2022; Anand et al., 2017). Studies have shown that individuals with these disorders often have higher levels of inflammatory markers in their blood compared to healthy individuals (Osimo et al., 2018). Additionally, treatments that target inflammation, such as anti-inflammatory medications, have shown promise in improving symptoms in some individuals with these disorders (Fitton et al., 2022). However, existing studies have mostly been conducted in adult populations, even though more than half of psychiatric disorders typically develop before the age of 18 years (Solmi et al., 2022). As such, further research is necessary to clarify the relationship between inflammation and psychiatric risk during early developmental stages. This includes identifying clinically relevant biomarkers for inflammation and considering key confounding factors to ascertain whether inflammation itself or another factor is driving these associations.

The perinatal period is crucial in the development of both the brain and the immune system (Han et al., 2021; Mwaniki et al., 2012). Moreover, inflammation during this susceptible period has been shown to associate with later neurodevelopmental outcomes (Jiang et al., 2018; Bangma et al., 2021). C-reactive protein (CRP) is an acute phase reactant to inflammation or infection and is widely used as a biomarker in both clinical and research settings. The advantage of CRP is that it can be easily measured with a reliable assay; however, the half-life of CRP is short (~9–12 h) (Ablij and Meinders, 2002). Hence, elevated levels of CRP after for example an acute infection or inflammation are rapidly restored to typical low levels. This means that CRP levels typically reflect a person’s inflammatory status temporarily, making single CRP measurements susceptible to misclassification bias for sustained inflammation. Ideally, to obtain a better characterization of sustained inflammation, repeated measurements of CRP would be needed. Yet, this is not always feasible in research setting and raises ethical concerns in vulnerable populations such as neonates. One strategy that has recently been proposed to address these challenges is to use an epigenetic proxy of CRP based on DNA methylation (DNAm) levels, derived from the results of a large-scale epigenome-wide association study (EWAS) of CRP, also referred to as methylation profile score, or MPS, akin to polygenic scores in genetics (Ligthart et al., 2016).

DNAm is an epigenetic mechanism that involves the addition of methyl groups to specific DNA base pairs, typically in the context of CpG dinucleotides, and can functionally alter gene expression. DNAm may represent a molecular pathway through which environmental and genetic factors influence neurodevelopment and psychiatric risk (Barker et al., 2018), as well as showing promise as a biomarker for risk detection and stratification (Lyskjaer et al., 2021). In the context of inflammation, multiple studies have shown that a DNAm profile score that proxies CRP levels (MPS-CRP) in blood and buccal saliva associates with immune-related disorders in adults, levels of maternal immune activation during pregnancy, and birth complications in preterm children, which in turn may underlie vulnerability for psychiatric conditions (Barker et al., 2018; Ligthart et al., 2016; Wielscher et al., 2022; Stevenson et al., 2020; Verschoor et al., 2023; Green et al., 2021). Moreover, a longitudinal study in adults showed that serum CRP levels were associated with changes in DNAm that were measured ten years later (Myte et al., 2019). Together, these findings suggest that MPS-CRP may act as a more stable proxy of sustained inflammation, which may help to capture cumulative inflammatory exposures at a molecular level (i.e., epigenetic ‘record’ of infectious or inflammatory insults).

Most studies on the relation of the MPS-CRP to inflammatory exposures and health outcomes focus on adults, leaving its utility in pediatric contexts largely unexplored, with only two notable exceptions. A recent study by Conole et al. (2023) examined MPS-CRP (derived from DNAm levels in saliva) in a group of preterm infants (N=155) versus term infants (N=103) (Conole et al., 2023). The authors found that MPS-CRP adequately captured inflammatory conditions such as chorioamnionitis or neonatal sepsis and that in turn, this score associated with adverse brain outcomes in infants, including lower total gray and white matter volume, as well as lower hippocampus and amygdala volume (Conole et al., 2023). Notably, associations were mainly limited to preterm infants, and not observed in infants born at term (N=103). With regard to behavioral and psychiatric outcomes, one other study in a population-based sample of 785 children (predominantly born at term) observed that MPS-CRP at birth indirectly associated with later higher internalizing and externalizing symptoms (at ages 7–15 years), as this was mediated by poorer cognitive function in childhood (Barker et al., 2018).

While these studies provide promising preliminary evidence for the use of MPS-CRP as a marker of neonatal inflammation and related neurobehavioral risk, several important gaps remain. First, no study has yet validated MPS-CRP against serum CRP levels at birth. This is important, given that MPS-CRP was developed based on analyses in adult blood and it is unclear to what extent it represents a good proxy of CRP in *cord* blood – often the first available tissue for assessing neonatal inflammation immediately following the intrauterine period and delivery, and differing from cord blood in cell-type composition and immune properties. Second, little is known about which factors associate with individual differences in MPS-CRP at birth – such as maternal prenatal lifestyle factors or health conditions – which could potentially be targeted to reduce inflammatory risk in offspring. Third, while the study by Conole et al. (2023) found that neonatal MPS-CRP associated with brain dysmaturity in 103 preterm infants (as reflected by lower white matter volume and connectivity) (Conole et al., 2023), it is unclear why effects were only observed in preterm infants; that is, whether this may be due to specific vulnerabilities in this group or potentially low statistical power to detect more subtle associations in term children. Studies are needed to better understand the relationship between MPS-CRP at birth and brain outcomes in the general pediatric population. Fourth, although one study reported evidence of indirect associations between MPS-CRP at birth and psychiatric problems in later childhood, it is unclear how this relationship may unfold over time: in other words, whether MPS-CRP shows persistent or waning associations with behavioral outcomes across development, which is important for assessing its potential value as a risk marker. Finally, previous neonatal studies computed the MPS-CRP based on seven probes identified by the EWAS of Ligthart et al. in 2016 (Ligthart et al., 2016). However, a larger, more recent EWAS by Wielscher et al. (2022; also performed in adult blood) identified over 1,500 probes significantly associated with serum CRP levels (Wielscher et al., 2022). Currently, it is not known how a more comprehensive MPS-CRP derived from these newer results performs in neonates, and to what extent it associates with prenatal predictors and neurobehavioral outcomes across development.

To address these gaps, we used data from nearly 2,400 mother–child dyads to comprehensively investigate predictors and outcomes of neonatal inflammation, as indexed by MPS-CRP at birth, within the general pediatric population. With this preregistered study, our goal was to better understand which prenatal factors explain individual differences in MPS-CRP at birth, and in turn, how this marker relates to long-term neurobehavioral outcomes, leveraging repeated measures data about behavior and brain from toddlerhood to early adolescence. Additionally, given previous findings, we investigated whether gestational age acts as a moderator that exacerbates the association between inflammation and neurobehavioral outcomes (Karahoda et al., 2021; El Marroun et al., 2020).

## 2. Methods

The analysis plan for this project was preregistered prior to any analyses (https://osf.io/utf8r). Minor deviations from the preregistration can be found in Supplementary Text A.

### 2.1. Study selection and participants

This project was conducted within the Generation R Study – a large prospective population-based cohort investigating health and development from fetal life onwards (Kooijman et al., 2016). To be eligible for this study, pregnant individuals had to have a delivery date between April 2002 and January 2006 and live in Rotterdam, the Netherlands. The Medical Ethics Committee of Erasmus MC, University Medical Center Rotterdam, approved all study procedures. All parents provided written informed consent. Children provided assent and from the age of 12 onwards they also provided informed consent.

To be included in our study, participants had to have (1) information on DNA methylation in cord blood at birth, (2) for aim 2 (see statistical analysis section below), one of the child brain outcomes had to be available at least at one of the two timepoints and (3) for aim 3 (see statistical analysis section below), one of the child behavior outcomes had to be available at one of the five timepoints. We excluded all twins in our study. We further excluded one sibling for each pair based on data completeness. If this information was equal, then we randomly excluded one sibling. Additionally, only for aim 2, we further excluded children with unusable or poor-quality MRI scans (e.g., due to incidental findings or major artefacts). DNA methylation data was only available for participants with European ancestry.

### 2.2. Assessment of methylation profile score of C-reactive protein (MPS-CRP)

Umbilical cord blood was drawn and DNAm profiles were then generated either with the Illumina Infinium HumanMethylation450 BeadChip array (Illumina Inc., San Diego, CA) (GenerationR_450k_: n = 1,367) or with the Illumina MethylationEPIC 850 K array (Illumina Inc., San Diego, CA) (GenerationR_EPIC_: n = 971; no overlapping samples between arrays). We used normalized, untransformed beta values that ranged from 0 (i.e. fully unmethylated) to 1 (i.e. fully methylated) to indicate methylation levels. Supplementary text B describes the sample processing, quality control and normalization steps in detail.

To construct MPS-CRP, we applied a method akin to that used for the calculation of polygenic risk scores. Specifically, we selected DNA methylation probes (CpGs) that have been previously found to associate with serum CRP levels in published epigenome-wide association studies (EWASs, see below), after which we multiplied the methylation beta value at each probe by the corresponding regression betas (i.e., weights) from the EWAS. Then, we summed these weighted methylation values into a single MPS-CRP. The scores were computed regardless of assay (450 K and EPIC) and standardized to Z-scores to enable comparison. As such, MPS-CRP represents a weighted aggregate score of CpGs previously found to associate with CRP levels. Weights allow us to ensure that the probes keep their relative magnitude of association with CRP based on independent discovery data.

Our main MPS-CRP score was calculated based on seven probes that have been found to associate most strongly to CRP as well as inflammation-related conditions in the EWAS by Ligthart et al., 2016 performed in adults and based on whole blood (discovery sample size = 8,863, Tables S1–S2) (Ligthart et al., 2016). This MPS has been previously utilized in a pediatric context, showing for example associations with inflammatory exposures and neurobehavioral outcomes in neonates (Barker et al., 2018; Conole et al., 2023). As a sensitivity analysis, we also calculated an extended MPS-CRP (MPS-CRP_extended_), based on 1,511 CpG sites recently identified to associate with CRP within a larger adult multi-cohort EWAS of CRP, also based on whole blood, (Wielscher et al., 2022; discovery sample size = 22,774, Tables S3–S4) (Wielscher et al., 2022). This score was previously found to associate with markers of inflammation and clinical phenotypes (e.g., cardiometabolic traits, lung function) in adult samples (Wielscher et al., 2022; Verschoor et al., 2023), but has not yet been applied to neonates. There is one previous EWAS of CRP that is based on cord blood (Yeung et al., 2020); however, Generation R was a large part of the discovery sample (~35 %) and therefore we could not use this EWAS due to non-independence of the CpG weights to build the score.

### 2.3. C-reactive protein measures in serum

To validate the MPS-CRP, we examined its associations with available serum high-sensitivity CRP (hs-CRP) in cord blood, which corresponds to the same tissue and time point. For comparative purposes, we also examined associations between the MPS-CRP and serum CRP levels measured at other available time points, including prenatal levels in mothers (in early and mid-pregnancy) and childhood levels in offspring, using different models for each CRP measurement. Distribution plots for the serum CRP variables can be found in Fig. S1. Maternal venous blood samples were collected during early and mid-pregnancy (mean age 13 weeks and mean age 25 weeks). Venous umbilical cord blood sampling was conducted immediately after delivery by midwives and obstetricians (Jaddoe et al., 2007; de Jonge et al., 2011). Child venous blood samples were collected at child mean age 5 years. These blood samples were then transported to the regional laboratory and stored at −80 °C. hs-CRP concentrations were measured in EDTA plasma samples at the Department of Clinical Chemistry of Erasmus MC. hs-CRP levels were analyzed using an immunoturbidimetric assay on the Architect System (Abbot Diagnostics BV, Hoofddorp, the Netherlands).

### 2.4. Assessment of clinical inflammatory scores

We constructed multiple prenatal clinical inflammatory scores as potential predictors of the MPS-CRP, namely: maternal prenatal infection, prenatal stress, lifestyle pro-inflammatory factors, pregnancy-related inflammatory conditions, and inflammatory medical conditions (Figs. S2–S3).

#### Prenatal infection:

infections were measured using an existing score, which has been previously constructed in Generation R (Suleri et al., 2023; Suleri et al., 2023), based on questionnaire data collected at three time points during pregnancy (once in approximately each trimester). In brief, women were asked to report on upper respiratory infections, lower respiratory infections, gastrointestinal infections, cystitis/pyelitis, dermatitis, eye infections, herpes zoster, sexually transmitted diseases, flu, and fever in each trimester of pregnancy. The presence/absence of each infection type were scored with 1 or 0 points, respectively. Scores were computed per trimester and then a mean score was calculated across pregnancy.

#### Prenatal stress:

we also used an existing cumulative score to assess prenatal stress, which has been previously constructed in Generation R based on questionnaire data (Bolhuis et al., 2022). Specifically, this score included 52 stress-related items that were selected and categorized as absent (0) or present (1) risks, and then summed into one of four domain scores: life events, contextual risk, parental risk, and interpersonal risk. These domain scores were then combined in a single cumulative prenatal stress score. More information can be found on: https://github.com/SereDef/cumulative-ELS-score.

#### Lifestyle pro-inflammatory factors:

we created a cumulative score for lifestyle pro-inflammatory factors, based on the following indicators that were measured with questionnaire data at enrollment: pre-pregnancy obesity (body mass index [BMI] > 30 [yes/no]) (Febbraio, 2014), any maternal tobacco use before or during pregnancy [yes/no] (Lee et al., 2012), maternal diet [food frequency questionnaire sum score (cut-offs based on above/below third quartile)] (Han et al., 2021), maternal psychotropic medication use during pregnancy (includes any use of selective serotonin reuptake inhibitors [SSRI], triptanes, antipsychotics, and tricyclic antidepressants [TCA] [yes/no]) (Yuan et al., 2019), maternal anti-inflammatory or anti-viral or bacterial infection medication use during pregnancy (includes any use of non-steroidal anti-inflammatory drugs [NSAIDs], antibiotics, paracetamol, mucolytics, antitussives [yes/no]), any type of maternal corticosteroid use during pregnancy [yes/no]). The presence of each inflammatory factor was scored with a point and the absence of a factor was given zero points.

#### Pregnancy related inflammatory conditions:

we created a cumulative score for pregnancy related inflammatory conditions, using the following indicators based on medical records: pre-eclampsia [yes/no] (Catarino et al., 2012), pregnancy-induced hypertension [yes/no] (Catarino et al., 2012), gestational diabetes [yes/no] (Dincer et al., 2022), premature ruptured membranes [yes/no] (Karahoda et al., 2021), HELLP syndrome [yes/no], caesarian delivery [yes/no] (Kiilerich et al., 2021). The presence of each condition was scored with a point and the absence of a condition was given zero points.

#### Inflammatory medical conditions:

we created a cumulative score based on the following indicators that were measured with questionnaire data at enrollment: diabetes [yes/no], intestinal disorder [yes/no], arthritis [yes/no], multiple sclerosis [yes/no], and thyroid disorder [yes/no]. The presence of each inflammatory condition was scored with a point and the absence of a condition was given zero points.

### 2.5. Polygenic score of CRP

Offspring genotype data were acquired in two different subsamples either from cord blood or through venipuncture during a visit to the research center at child mean age 6 years if cord blood was not available, utilizing the 610 K or 660 K SNP array by Illumina in San Diego, CA. Details regarding sample collection and genotype calling methodologies have been outlined elsewhere, as have quality control procedures, genotype imputation, and principal component calculation (Jaddoe et al., 2007; Bolhuis et al., 2022; Medina-Gomez et al., 2015; Jansen et al., 2019). For the polygenic score (PGS) calculation, we utilized genotype data imputed to the 1,000 genomes reference panel (phase 3 version 5) retaining SNPs with an imputation quality >=0.8 and a minor allele frequency >=0.01 and excluding multi-allelic and duplicate SNPs. The script for this can be found: https://github.com/inDEPTHlab/PRS/blob/main/vcf_conversion/readme_bed_rds_files.md.

To construct a PGS of CRP we used a recent genome-wide association study (n = 575,531) (Said et al., 2022). Summary statistics were obtained from the GWAS catalog (ID = GCST90029070). We used the LDpred2 function from the ‘bigsnpr’ package in R to calculate the PGS using HapMap3 variants with independent LD blocks (Privé et al., 2021). After calculating the PGSes in each subsample, we residualized the first ten genetic principal components on the *Z* scored PGS to standardize across subsample and control for batch effects, before merging these together. A higher PGS score signifies a higher genetic predisposition for elevated CRP levels. Previous research has demonstrated that the PGS of CRP correlates with health outcomes and exhibits strong performance across various adult cohorts with serum CRP (Kappelmann et al., 2021; Aday et al., 2023).

### 2.6. Assessment of child brain morphology and white matter microstructure

We used both structural MRI as well as DTI data at the mean ages of 10 and 14 years to extract the following outcomes: (1) structural MRI: volumes of total brain, total gray matter, total white matter, cerebellum, hippocampus, amygdala, brain stem and lateral ventricles (no lateralized hypothesis; hence, we averaged across both hemispheres), as well as (2) DTI: global fractional anisotropy and global mean diffusivity (a weighted average of all the tracts).

#### 2.6.1. Image acquisition

We used T1-weighted structural MRI images to obtain measures of brain morphology and DTI to obtain information on white matter microstructure. All children at each time point were offered to participate in a mock MRI scanning session to become familiar with the MRI procedure (White et al., 2018). For both modalities, a 3-Tesla GE Discovery MR750w (GE, Milwaukee, WI) system was used with an 8-channel head coil. (Dall’Aglio et al., 2023). High resolution T1-weighted scans were obtained with an IR-FSPGR sequence with the following parameters: repetition time = 8.77 ms, echo time = 3.4 ms, inversion-time = 600 ms, number of excitations = 1, flip angle = 10°, matrix size = 220×220, ARC imaging acceleration factor = 2, and the isotropic resolution = 1.0 mm^3^ (White et al., 2018). The DTI measurement included a 35-direction echo planar imaging sequence, with the following parameters: repetition time = 12,500 ms, echo time = 72 ms, field of view = 240 mmx240 mm, acquisition matrix = 120×120, slice thickness = 2 mm, slices = 65, ASSET acceleration factor = 2, b = 900 s/mm^2^, 3b = 0 images (Dall’Aglio et al., 2023).

#### 2.6.2. Image processing

sMRI data were processed with the FreeSurfer analysis suite (Muetzel et al., 2019). DTI data were processed with a fully automated probabilistic fiber tractography from which subject-specific probabilistic representations of different white matter fibers bundles were obtained (FSL plugin ‘AutoPtx’ (http://fsl.fmrib.ox.ac.uk/fsl/fslwiki/AutoPtx) (Muetzel et al., 2018; Dall’Aglio et al., 2022). Data underwent quality control with various stages of visual inspection which were complemented with automated assessments. A brief summary can be found in Supplementary text C and full details on the quality assurance for both modalities can be found elsewhere (White et al., 2018; Muetzel et al., 2019; Muetzel et al., 2018; Dall’Aglio et al., 2022).

### 2.7. Assessment of child psychiatric symptoms

Child psychiatric symptoms were assessed with parent-reported ratings on the Child Behavior Checklist (CBCL) (Achenbach and Rescorla, 2001). We administered the CBCL repeatedly from mean ages of 1.5 (=T1), 3 (=T2), 6 (=T3), 10 (=T4), and 14 (=T5) years using the CBCL version 1.5–5 (T1-T3) and version 6–18 (T4, T5). The CBCL version 1.5–5 years consists of 99 items and the CBCL version 6–18 years consists of 112 items. Both versions are reliable and valid questionnaires and thus widely used for assessing common emotional and behavioral problems (Achenbach & Rescorla, 2000, 2001). Psychometric studies have demonstrated that broad symptoms of mental health and neurodevelopment can be grouped into internalizing and externalizing syndromes throughout childhood (Achenbach, 1991). Here, we used a sum score for the broadband scales: total psychiatric symptoms, internalizing (emotional) problems, and externalizing (behavioral) problems, in which higher scores indicate greater problems.

### 2.8. Assessment of childhood inflammatory outcomes

When the child was 13–16 years old, we also measured height (m) and weight (kg) at the research center, after which BMI of the child was calculated. Subsequently, BMI-SDS was computed, adjusting for age and sex according to Dutch reference growth curves (Talma et al., 2010). Moreover, parent-reported questionnaire data was collected during this research wave to ask whether the child was previously diagnosed with asthma [yes/no], ever used medication for allergy related symptoms [yes/no], or ever experienced eczema [yes/no].

### 2.9. Covariates

We adjusted for a range of covariates, as indicated per model in the analysis section and described below: namely maternal age at enrollment, maternal tobacco use (‘no’, ‘yes, until pregnancy was known,’ and ‘yes, continued during pregnancy’), parity, household income (‘<€2,200 per month’ or ‘>€2,200 per month’; cut-off based on the net average income of 2002) and maternal education were prospectively assessed with a questionnaire at enrollment. Following the Statistics Netherlands classification, three categories were created for maternal education: ‘primary’ (no education or primary school), ‘intermediate’ (secondary school or lower vocational training) and ‘high’ (higher vocational training or university). Moreover, gestational age at birth (weeks) and child sex were obtained from medical records. Child age was established from the date of birth and the date of questionnaire completion. In all analyses that included the MPS-CRP, sample plate was included as a technical covariate to adjust for batch effects and we used a cord-blood reference panel to adjust for cell type proportions (Gervin et al., 2019). Intracranial volume (ICV) was measured with structural MRI. Fig. S4 shows a Directed Acyclic Graph (DAG) for the covariates. Fig. S5 shows the data collection time point for all study variables.

### 2.10. Statistical analyses

**Fig. 1.**
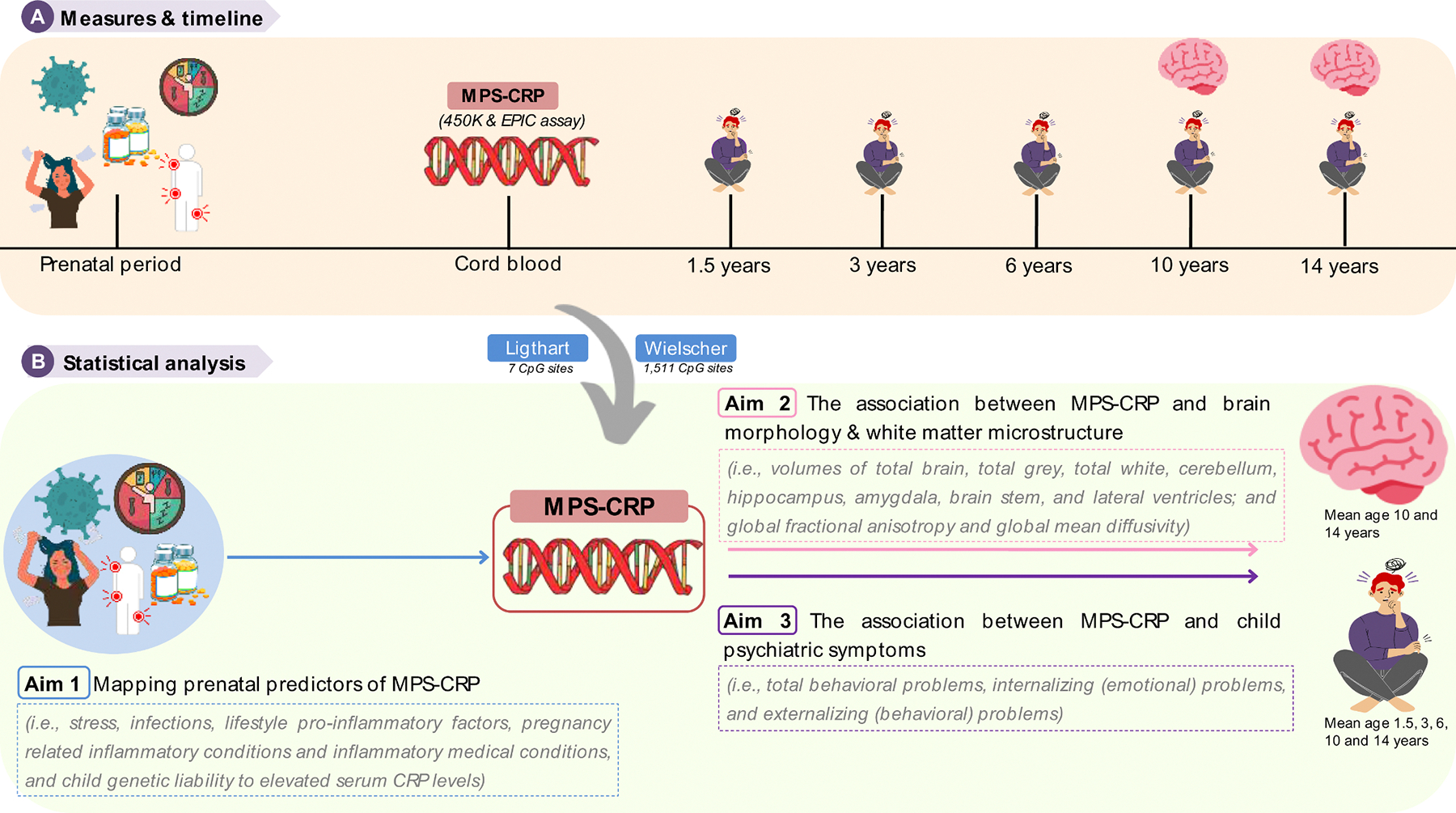
Statistical analyses overview.

All analyses were performed in R statistical program version 4.1.2 (R Core Team, 2021). A visual overview of the three aims and statistical analyses of our study can be found in Fig. 1 and are described in detail below. The analytical code used for this project can be found on https://github.com/ajsuleri/MPS_neonatal_CRP_neurodevelopment.

Analyses were conducted separately for each subcohort based on DNAm array (GenerationR_450k_, GenerationR_EPIC_), after which the results were *meta*-analyzed with inverse variance weighted fixed-effects *meta*-analysis using the ‘metafor’ package (Viechtbauer, 2010). An alpha level of 0.05 was used to assess statistical significance, and when applicable, multiple testing correction, within each aim and within each model but across all outcomes, was applied using the false discovery rate – Benjamini Holchberg (FDR-BH) correction (Benjamini and Hochberg, 1995), where q < 0.05 was interpreted as statistically significant.

The main analyses were performed using MPS-CRP based on the Ligthart et al. EWAS (Ligthart et al., 2016), to maximize comparability with previous pediatric studies. For completeness and comparative purposes, analyses were repeated with an extended MPS-CRP based on a more recent and larger CRP EWAS (Wielscher et al., 2022).

#### 2.10.1. Validation of MPS-CRP with serum CRP

To validate MPS-CRP in cord blood, we mapped correlations and performed receiver operating characteristics curve [ROC] analyses using measures of serum CRP available at different time points in Generation R, namely: (1) CRP assessed in *cord blood at birth* (caveat: CRP levels are at the lowest detectable limit in 94 % of this sample, suggesting the possibility of measurement error); (2) CRP in whole blood *at child aged 5 years* (caveat: not the target tissue/age), and (3) maternal *prenatal* CRP levels in trimester 1 and 2 (caveat: maternal CRP levels may not reflect neonatal CRP levels). Due to the limitations of cord blood, we included serum CRP measurements at various time points to validate MPS-CRP as thoroughly as possible. We further tested the correlation between the main MPS-CRP (i.e., based on Ligthart et al., 2016; seven CpGs) and the MPS-CRP_extended_ (based on Wielscher et al., 2022; 1,511 CpGs).

#### 2.10.2. Aim 1: Mapping prenatal predictors of MPS-CRP

To investigate our first aim, i.e., to examine which prenatal factors drive MPS-CRP, we conducted a multivariate linear regression model. The following prenatal factors were entered jointly in one model as predictors: stress score, infection score, lifestyle inflammatory factors, pregnancy related inflammatory conditions, and inflammatory medical conditions. Furthermore, we ran a separate linear regression model where we associated PGS of CRP (exposure) to MPS-CRP (outcome). The model with PGS of CRP was run individually because prenatal risk factor scores might act as mediators in the pathway, potentially causing overadjustment. In all the models, we adjusted for child sex, batch effects, estimated cell type proportions, household income, and maternal education.

#### 2.10.3. Aim 2: Mapping brain outcomes of MPS-CRP

To investigate our second aim, i.e., to study the association between MPS-CRP and child brain morphology and white matter microstructure, we used linear mixed-effects models (‘lme4′ and ‘broom.mixed’ package) (Bates et al., 2015). We used imaging data at two time points (child mean ages 10 and 14 years) and examined the brain morphology and white matter microstructure outcomes in individual models. We conducted three models, all of which included a random intercept for participant ID to account for repeated observations per person, enabling us to examine: (1) the average association between MPS-CRP and child brain morphology and white matter microstructure over time (brain outcome ~ MPS-CRP+child age + covariates), (2) the association between MPS-CRP and *changes* in child brain morphology and white matter microstructure over time, to understand if there is an age-dependent association (brain outcome ~ MPS-CRP * child age + covariates), and (3) the association between the three-way interaction of MPS-CRP, time and gestational age at birth (brain outcome ~ MPS-CRP * child age * gestational age at birth + covariates) on child brain morphology and white matter microstructure, to establish whether MPS-CRP associates with child brain morphology and white matter microstructure differently over time based on gestational age at birth. Covariates were as per aim 1 with additional adjustment for maternal age at delivery, maternal smoking during pregnancy, parity, and the child’s age (as time variable at each measurement moment). As a specificity analysis, we additionally adjusted for ICV in the first two models with a brain morphology outcome.

#### 2.10.4. Aim 3: Mapping behavior outcomes of MPS-CRP

For our third aim, i.e., examining the association between MPS-CRP and child psychiatric symptoms, we applied linear mixed-effects models. We used data at 5 different time points (child mean ages 1.5, 3, 6, 10, and 14 years) and examined each behavior outcome (internalizing, externalizing and total behavioral problems) separately. Similarly to aim 2, we conducted three models, all of which included a random intercept for participant ID to account for repeated observations per person, in order to examine: (1) the average association between MPS-CRP and child psychiatric symptoms over time (behavior outcome ~ MPS-CRP+child age), (2) the association between MPS-CRP and *changes* in child psychiatric symptoms over time (behavior outcome ~ MPS-CRP * child age), (3) the three-way interaction of MPS-CRP, time and gestational age at birth (behavior outcome ~ MPS-CRP * child age * gestational age at birth) on child psychiatric symptoms. Multiple testing correction within each model was applied using the false discovery rate – Benjamini Hochberg (FDR-BH) correction (Benjamini and Hochberg, 1995), where q < 0.05 was interpreted as statistically significant. Of note, the CBCL outcomes were square root transformed to improve adherence to the normality of residuals assumption. Covariates were as per aim 2.

#### 2.10.5. Sensitivity analyses

We conducted three sensitivity analyses. First, because cell type proportions may also be affected by inflammation status at birth and could therefore represent mediators, we repeated analyses without adjusting for estimated cell type proportions. Second, as a sensitivity analysis to aim 3 (association between MPS-CRP at birth and mental health outcomes in offspring), we were also interested whether the MPS-CRP at birth associates with physical (i.e., inflammatory) outcomes in offspring (mean age 14 years). We were specifically interested in asthma, allergy, eczema, and SDS-BMI. We performed logistic regression models for asthma, allergy and eczema, and linear regression models for SDS-BMI. Third, we reran all the analyses using the MPS-CRP_Extended_ score, to test whether an epigenetic score based on a larger and more recent adult EWAS of CRP (containing a wider set of CpG sites), which has not yet been used in a pediatric context, shows consistent associations with prenatal factors and neurodevelopmental outcomes compared to our main MPS-CRP.

#### 2.10.6. Missing data

To account for missing data, we performed multiple steps. First, for the construction of MPS-CRP score, we imputed missing CpG sites at an individual level using k-nearest neighbors with the ‘impute’ package (maximum percent missing data allowed in any row and column was 50 % and 80 %, respectively) (Trevor Hastie, 2017). If a CpG site was missing across all participants, we did not impute it. Moreover, we imputed missing covariates, CBCL scores, and the item level data for the prenatal predictor scores with multiple imputation by chained equations (30 datasets and 60 iterations) in the’mice’ package (Buuren et al., 2011), before passively deriving the prenatal predictor scores. We did not impute MPS-CRP or brain imaging data.

## 3. Results

### 3.1. Descriptive results

**Fig. 2.**
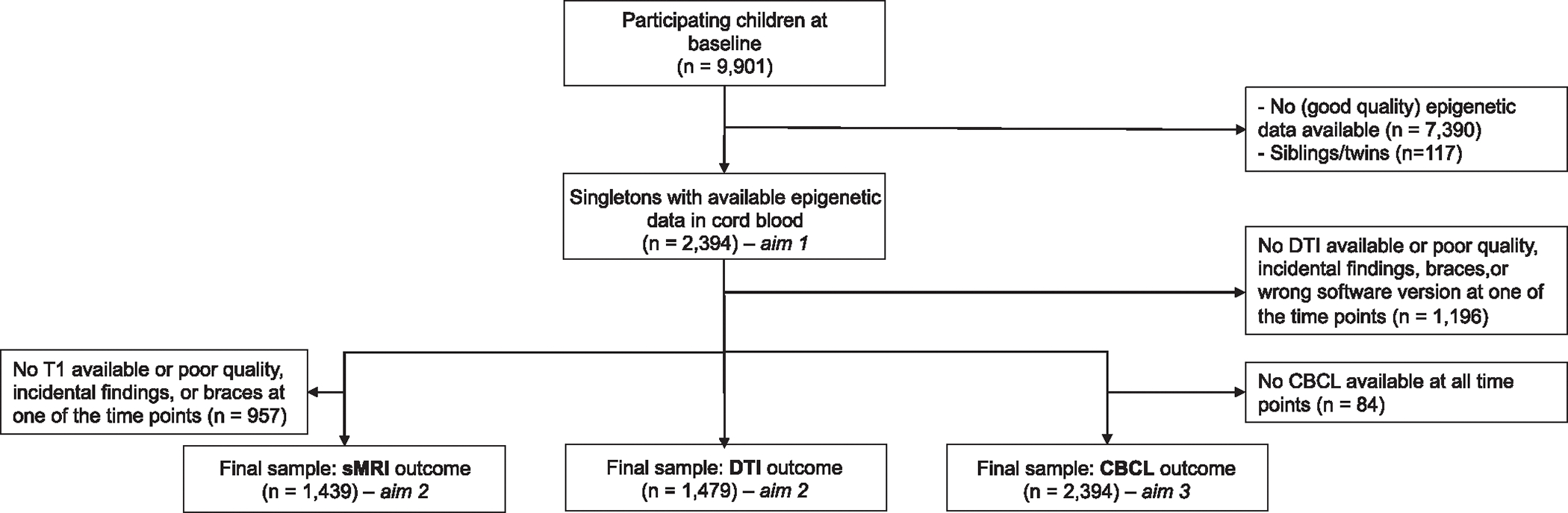
Flowchart depicting selection of study participants.

A total of 2,394 mother–child dyads were eligible for inclusion in our study for aim 1 and 3 (Fig. 2) and a total of 1,439 and 1,479 mother–-child dyads were eligible for aim 2 (sMRI and DTI samples, respectively; Fig. 2). Baseline characteristics of the sample can be found in Table 1. A correlation plot of all included variables can further be found in Fig. S6.

### 3.2. Validation of MPS-CRP with serum CRP

The MPS-CRP showed a normal distribution (Fig. S7). ROC curves of MPS-CRP and serum CRP in cord blood (clinical cut-off > 1 mg/L as indicator of neonatal infection) (Edgar et al., 2010; Kordek et al., 2008) showed an area under the curve (AUC) of 0.76 and 0.68 in the GenerationR_450K_ and GenerationR_EPIC_ samples, respectively (Fig. S8). Consistent with this, MPS-CRP showed a nominally significant association with elevated levels of CRP in cord blood based on univariate (β = 0.046, SE = 0.023, p = 0.049) and batch effect-adjusted models (β = 0.071, SE = 0.026, p = 0.007) (Table S5, Fig. S9). However, this association was no longer significant in the model that was additionally adjusted for cell type proportions (β = 0.072, SE = 0.038, p = 0.057) (Table S5, Fig. S9). When interpreting these results, it is important to note that there is a major skew in the data, with 94 % of the CRP data in cord blood being below the limit of detection (Figs. S1, S8). MPS-CRP did not associate with serum CRP levels from the mother in pregnancy (trimester 1 and 2) or CRP levels from the offspring at a different age (5 years), indicating some degree of specificity to CRP levels in cord blood (i.e., the target tissue and time point; Table S5).

Univariate regressions between serum CRP levels in cord blood and the individual CpG sites from the original EWAS for the main MPS-CRP, 2/5 CPG sites showed a significant association after multiple testing correction (Table S2). Of note, these results are based on the CpG sites available in both 450 K and EPIC arrays, whilst the MPS scores used in this paper are derived from the maximum number of CpG sites available for each array.

### 3.3. Aim 1: Mapping prenatal predictors of MPS-CRP

**Fig. 3.**
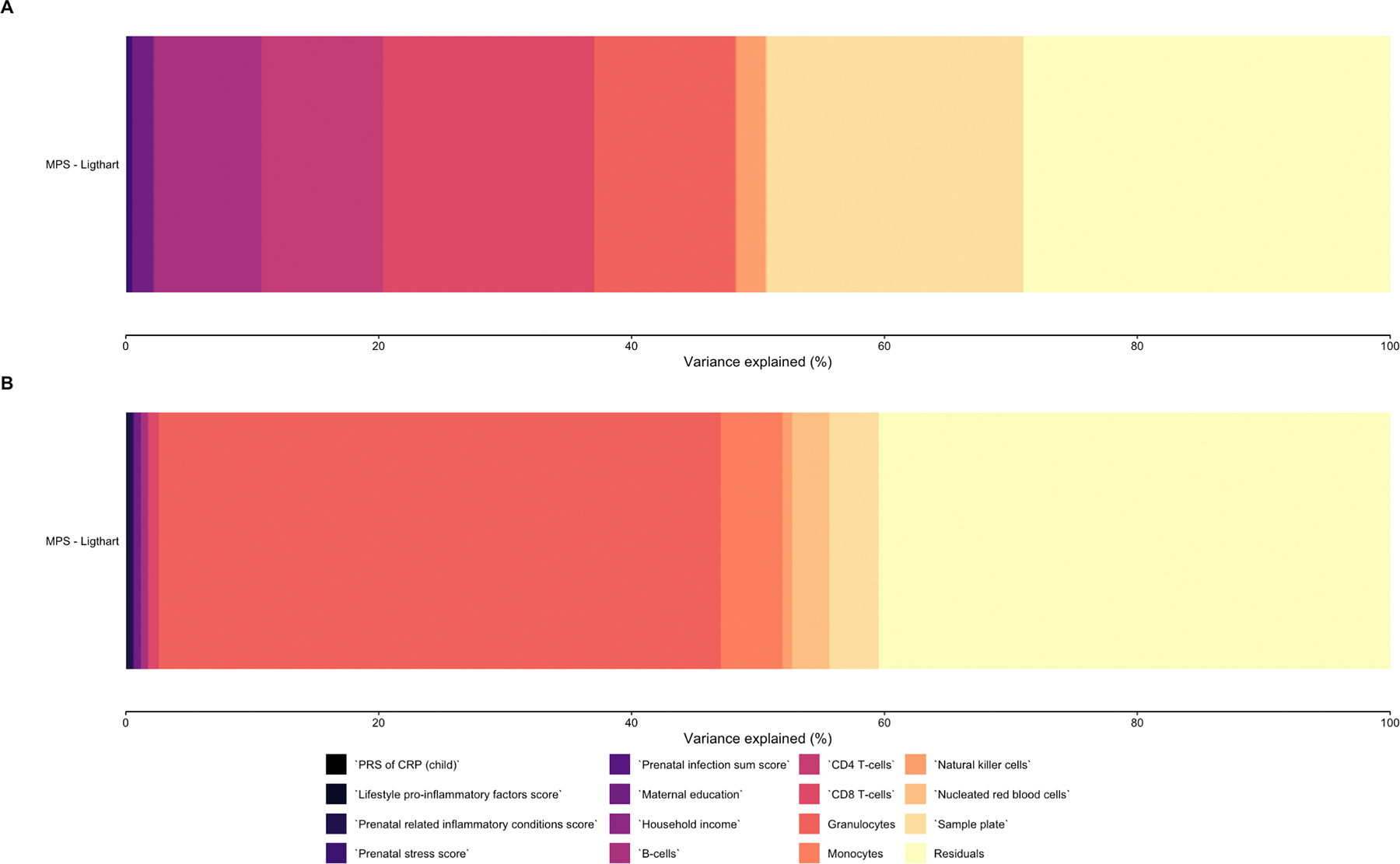
Variance partition analysis for aim 1. This plot displays the variance explained in the MPS by various prenatal factors, the PGS of CRP, cell type proportions, and residuals, for the 450 K array and EPIC array separately.

**Fig. 4.**
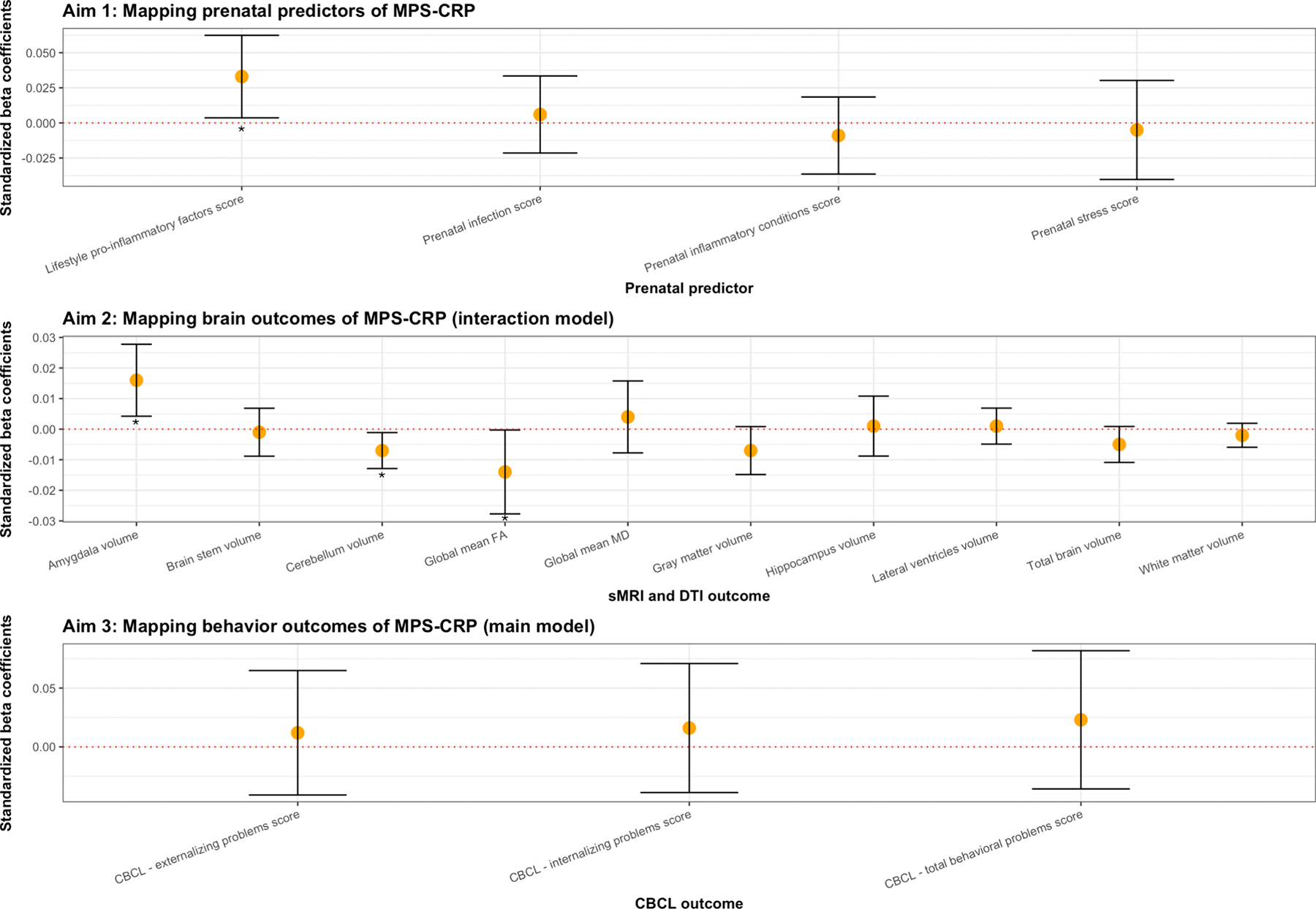
Primary analyses results for each aim (for MPS-CRP_Ligthart_). A single asterisk indicates p < 0.05. The error bars show the 95 % confidence interval.

Prenatal exposure to lifestyle pro-inflammatory factors showed a nominally significant, albeit small, positive association with elevated MPS-CRP at birth (β = 0.033, SE = 0.015, p = 0.027), over and above other prenatal maternal factors. No other prenatal maternal factor was independently associated with MPS-CRP at birth (Table 2, Figs. 3, 4 and S10).

PGS of CRP associated with serum CRP measures in the mother and in the child (*ρ*_spearman_ = 0.21–0.23, p < 0.05), but not in cord blood (*ρ* spearman = 0.04, p > 0.05) (Table S6, Fig. S11). We found no evidence for a significant association between PGS of CRP in the child and MPS-CRP at birth (Table S7). Overall, variance partition analysis showed that estimated cell type proportions were the largest driver of the MPS-CRP (Fig. 3).

### 3.4. Aim 2: Mapping brain outcomes of MPS-CRP

**Fig. 5.**
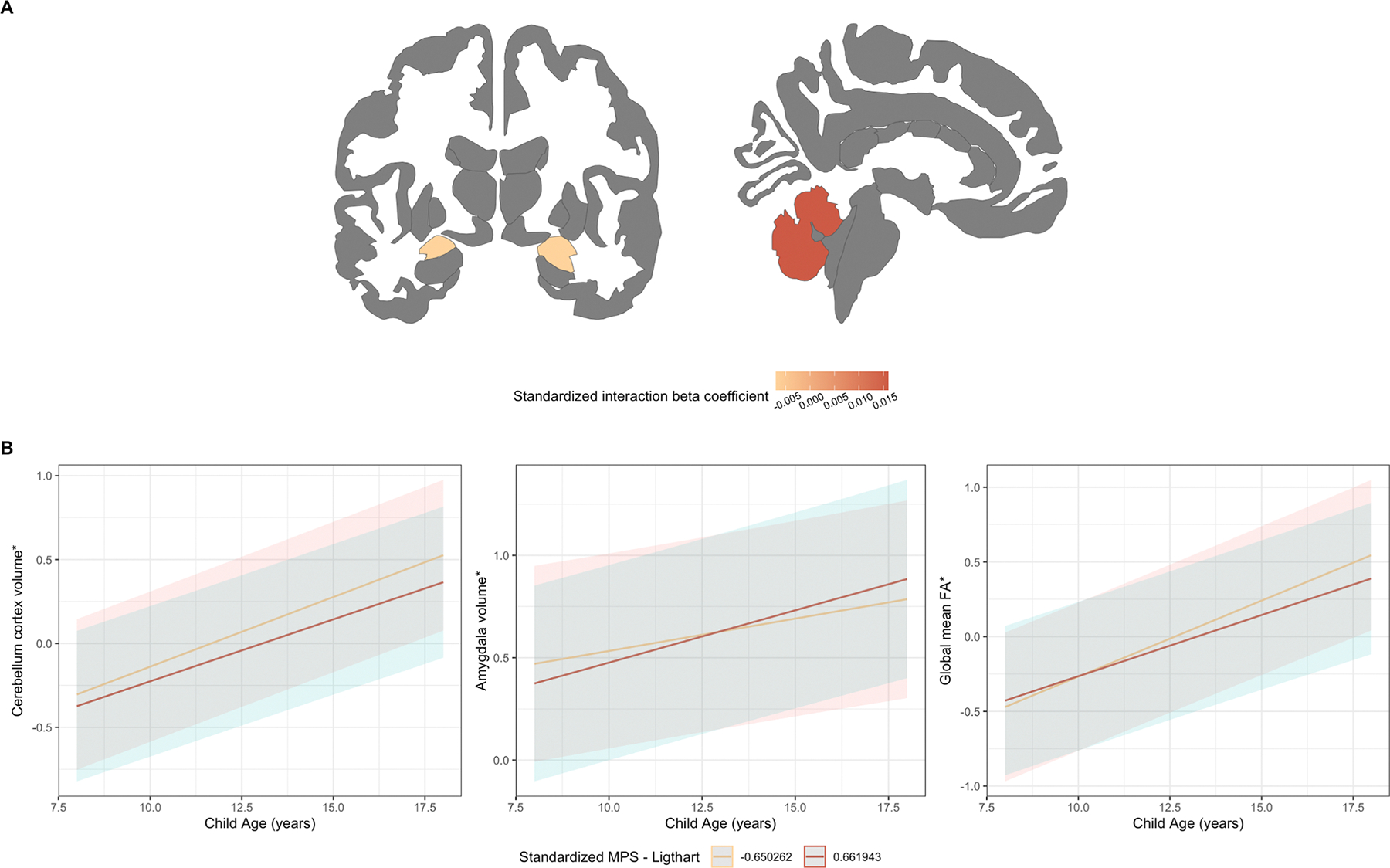
Suggestive findings for brain findings (aim 2). Fig. 5A depicts the effect size and location of the observed brain regions. Fig. 5B depicts the longitudinal trajectory for the 1st and 3rd quartile MPS-CRP group. Moreover, * Indicates p_uncorrected_ < 0.05.

MPS-CRP was nominally associated with an increase in amygdala volume (β = 0.016, SE = 0.006, p = 0.010), a decrease in cerebellar volume (β = −0.007, SE = 0.003, p = 0.036) and a decrease in global fractional anisotropy (β = −0.014, SE = 0.007, p = 0.032) over time (Table 3, Figs. 4–5, Fig. S12). None of these associations, however, survived multiple testing correction. Further, we observed no moderation effect of gestational age at birth. The direction of the results was comparable when additionally adjusting for ICV (Table S8).

### 3.5. Aim 3: Mapping behavior outcomes of MPS-CRP

MPS-CRP was not significantly associated with average internalizing, externalizing or total problem scores or their change over time in the main models (Table 4 and Fig. 4) or the sensitivity analyses (Table S9). Of note, because of the high correlation between the pregnancy related inflammatory clinical score and the medical inflammatory conditions score (r > 0.9), we omitted the medical inflammatory conditions score from the multivariate model.

### 3.6. Sensitivity analyses

#### 3.6.1. Cell type proportions

The results of the first sensitivity analysis, running the primary analyses for all outcomes without adjusting for cell type proportions can be found in the following tables: prenatal predictors = Table 2, brain outcomes = Table S8, behavior outcomes = Table S9). The sensitivity analysis showed that a higher score for inflammatory clinical conditions in pregnancy was associated with a small decrease in the MPS-CRP when cell heterogeneity was not taken into account. This highlights the potential influence of cell type composition on the relation between neonatal inflammation and CRP-related DNA methylation, which we controlled for in our main analyses.

#### 3.6.2. Childhood inflammatory outcomes

The results of the second sensitivity analysis, which examined the association between MPS-CRP at birth and inflammatory outcomes in childhood, indicated no association between MPS-CRP at birth (both the main and extended MPS) and any inflammatory outcomes after multiple testing correction (Table S10).

#### 3.6.3. Use of an extended MPS-CRP score

Despite including a much larger set of CpG sites (1,511 vs 7), the MPS-CRP_Extended_ score (Fig. S7) showed a strong, positive correlation with our main MPS-CRP score (*ρ*_pearson_ = 0.70, p < 0.05). Based on ROC curves, however, this extended score demonstrated poorer predictive performance than the main MPS-CRP in relation to serum CRP levels at birth (Fig. S11), with similar AUC values observed across arrays (GenerationR_450k_ = 0.55; GenerationR_EPIC_ = 0.53) (Fig. S8). Univariate regressions between serum CRP levels in cord blood and the individual CpG sites from the original EWAS for the extended MPS-CRP, 154/1401 CPG sites showed a significant association after multiple testing correction (Table S4). Of note, these results are based on the CpG sites available in both 450 K and EPIC arrays, whilst the MPS scores used in this paper are derived from the maximum number of CpG sites available for each array. Associations with predictors and neurodevelopmental outcomes were generally consistent in direction to MPS-CRP, but weaker in magnitude and non-significant (Figs. S13–15, Tables S11–13). The only exception was the association between MPS-CRP_Extended_ score and a decrease in global fractional anisotropy over time (β = −0.018, SE = 0.006, p = 0.005) (Fig. S14, Table S13), which remained significant after multiple testing correction.

## 4. Discussion

The aim of this study was to map prenatal predictors and neurobehavioral outcomes of neonatal inflammation, measured by a DNA methylation profile score as a proxy of CRP (MPS-CRP) at birth, using data from nearly 2400 mother-infant dyads from the general population. We highlight here our key findings. First, the MPS-CRP at birth showed adequate predictive performance in relation to serum CRP levels in cord blood (average AUC = 0.72), whereas it did not associate with either maternal CRP levels during pregnancy or offspring levels later in childhood, supporting a degree of specificity to neonatal CRP levels. Second, despite investigating a wide range of potential predictors of neonatal inflammation – including maternal prenatal stress, prenatal infections, lifestyle inflammatory factors, pregnancy-related inflammatory conditions, and child genetic predisposition for CRP levels – only prenatal lifestyle inflammatory factors showed a unique association with MPS-CRP at birth. Third, we found suggestive evidence of prospective associations between the MPS-CRP and lower white matter integrity – consistent with previous findings in pre-term infants (Conole et al., 2023), although this association only survived multiple testing correction in our MPS-CRP_Extended_ score. Third, no associations with behavior outcomes were identified. Finally, our sensitivity analysis showed that MPS-CRP at birth was not associated with inflammatory outcomes in childhood, such as asthma, allergy, eczema or BMI. Together, these findings cast some doubt on the potential utility of the MPS-CRP as a neonatal marker of inflammatory insults and neurobehavioral risk within the general population, although tentative evidence of associations with prenatal lifestyle factors and white matter disruptions are consistent with the broader literature and warrant further investigation. Weak associations may also suggest that inflammatory insults around delivery that are mild and detected on a population level may not have a pronounced impact on brain nor behavior later in life.

### 4.1. MPS-CRP as a marker of neonatal inflammation

In the field of epigenetic epidemiology, DNA methylation profile scores (MPSs) are increasingly showing promise as markers for disease stratification (e.g., MPS for BMI or accelerated ageing) as well as viable surrogate markers for exposures or outcomes that are not directly measured or difficult to assess reliably (e.g., smoking (Nabais et al., 2023)). In the context of inflammation, previous studies have shown that the MPS-CRP robustly associates with CRP levels as well as relevant health outcomes in child and adult cohorts (Ligthart et al., 2016; Barker et al., 2018; Wielscher et al., 2022; Stevenson et al., 2020; Green et al., 2021; Conole et al., 2023). However, its application to cord blood at birth – often the first available tissue for assessing neonatal inflammation – has not yet been validated. In this study, we found that the MPS-CRP shows adequate discriminative ability between high and low serum CRP levels in cord blood. Of note, performance was higher when the 450 k array was used to construct the score (AUC = 0.76) compared to the EPIC array (AUC = 0.68), which may be attributable to the discovery EWAS used to derive weights for the MPS, which only used 450 K data (Ligthart et al., 2016).

Interestingly, the MPS-CRP did not show any associations with other serum CRP measures available in our cohort, namely maternal CRP during pregnancy (i.e., different target individual; maternal levels may not reflect neonatal levels), and child CRP at age 5 years (i.e., same individual, but different tissue and time point). This supports a certain degree of specificity to CRP levels at birth, despite the low detectability compared to CRP levels in mothers during pregnancy or in offspring during childhood. It is important to note that very few neonates in our sample had serum CRP levels at birth above the detection limit (0.2 mg/L). This could be due to measurement error, the population-based nature of our sample (which primarily includes uncomplicated pregnancies with term infants), or both. The skewness in CRP measurements may have further influenced our results. Consistent with our findings, another population-based cohort, ALSPAC, reported a mean CRP level of 0.2 mg/L in cord blood (Hillary et al., 2024). In contrast, clinical samples have demonstrated that measuring CRP in cord blood can effectively predict neonatal complications such as sepsis and infections, as well as maternal complications like amniotic infection and funisitis (Yadav et al., 2023; Yoon et al., 1997; Joram, 2005; Kitano et al., 2019). This suggests that CRP measurement in cord blood might be more accurately performed in clinical high-risk settings rather than in general population samples. Together, these issues render the interpretation of our validation results challenging; in other words, are the weak correlations that we observed a sign that the MPS-CRP does not adequately capture neonatal inflammation, or that the serum CRP measurement at birth is unreliable (at least in the general population)? In the future, it may be worthwhile to explore alternative inflammatory markers, such as procalcitonin levels (Yadav et al., 2023), which have been reported to show superior performance in neonates compared to CRP levels in cord blood.

Furthermore, for comparison purposes, we also re-ran analyses using an extended MPS-CRP based on a larger set of DNA methylation sites (circa 1500 vs 7, with 6/7 CpGs overlap) identified in a more recent and powered EWAS of CRP (Wielscher et al., 2022). The two scores were highly correlated; yet the MPS-CRP_Extended_ showed lower predictive performance (comparable between arrays: AUC in 450 K = 0.55, AUC in EPIC = 0.53, potentially reflecting the use of both arrays in the discovery EWAS). This may suggest that the epigenetic signal of CRP at birth is better captured by a parsimonious score, with this signal becoming diluted with the use of a more comprehensive set of sites.

### 4.2. Prenatal predictors of MPS-CRP

After validating the MPS-CRP, our next step was to better understand which factors may explain variability in this score at birth within the general population. We examined a wide set of prenatal predictors previously linked to inflammation, aggregated into scores for maternal prenatal stress, prenatal infections, lifestyle inflammatory factors, pregnancy-related inflammatory conditions, totaling over 70 included variables. Nominal associations were identified between pro-inflammatory lifestyle factors (e.g., high BMI, poor diet, tobacco use or anti-inflammatory or psychotropic medication use) and the MPS-CRP, while controlling for the other scores. Given the use of an epidemiological sample, it is possible that the other factors investigated were either not severe enough (e.g., exposure to common infections such as influenza instead of severe infections), or their prevalence too low (e.g., pre-eclampsia) to explain individual differences in MPS-CRP at a population level. Furthermore, our cumulative approach (i.e., summing exposures into total scores) may have obscured associations if these are confined to (timing) specific exposures. Other unmeasured variables may also be relevant to MPS-CRP, such as those related to placental function and labor itself (Gomez-Lopez et al., 2014; Bollapragada et al., 2009). For example, placental conditions such as chorioamnionitis (inflammation of the fetal membranes), chorionic vasculitis (inflammation of the blood vessels within the chorionic plate of the placenta), funisitis (inflammation of the umbilical cord), and atypical placental microorganisms have all been linked to fetal inflammatory response syndrome (FIRS), and may thus play a significant role in driving inflammation in cord blood (Bangma et al., 2021; Gibson et al., 2023; Mestan et al., 2009), necessitating further investigation.

Besides prenatal factors, we did not find that the child’s genetic predisposition to elevated CRP levels predicted the MPS-CRP at birth. From studies performed in adults, it is evident that this PGS strongly associates with both blood serum CRP levels and downstream cardiovascular outcomes (Said et al., 2022). It is noteworthy that the PGS of CRP (calculated from child genetic data), did associate with maternal CRP levels (i.e., potentially reflecting shared genetic influences) as well as the child’s own CRP levels at age 5 years. This may be due to a noisier measurement of serum CRP at birth (i.e., most samples below detection limit) or potentially due to biological differences in expression and regulation of CRP between tissues (cord blood [at birth] versus whole blood [in mothers during pregnancy and in later childhood]). Additionally, it may be that other (genetic) factors contribute to CRP levels in cord blood (e.g., related to labor and fetal immune development) compared to later life (childhood or adulthood). Notably, variance decomposition analyses showed that most variance in MPS-CRP at birth was actually explained by estimated cell-type proportions. This occurrence may be largely attributed to these proportions predominantly reflecting the composition of white blood cells, which are important components of our immune system.

### 4.3. Neurobehavioral outcomes of MPS-CRP

Finally, we examined whether the MPS-CRP at birth prospectively associated with child brain and behavioral outcomes. With regards to the brain, we found suggestive (i.e., nominally significant) associations between the MPS-CRP at birth and alterations in white matter connectivity (DTI) as well as amygdala and cerebellum volume between childhood to adolescence (structural MRI). Of note, a significant association with reduced white matter connectivity − as indexed by lower global fractional anisotropy – was also identified when using the MPS-CRP_Extended_, which survived correction for multiple testing. White matter is important for coordinating communication between different brain regions via myelinated nerve fiber bundles that transmit electrical signals (Fields, 2010). This synchronization supports cognitive processes, integrates sensory and motor functions, and is involved in tasks such as memory and learning (Dubner et al., 2019). White matter microstructure frequently appears as one of the most robust findings in multiple preclinical and clinical studies of neonatal infection or inflammation, particularly among preterm infants (Anblagan et al., 2016; Basu et al., 2014; Gaudet et al., 2009; Nosaka et al., 2023; Shevell et al., 2014). Indeed, the previous study by Conole et al (Conole et al., 2023) using neonatal MPS-CRP found that it prospectively associated with lower white matter integrity, but only in preterm infants. It is possible that the use of a larger sample in our study (i.e., 1,479 predominantly term-born children for DTI analyses versus 103 preterm infants in the previous study) enabled us to detect more subtle associations present in the general population. Reasons for observing findings in white matter microstructure and not white matter volume may be due to the different nature of both structures. For example, microstructure is more sensitive to subtle alterations in the organization of white matter fibers (e.g., damage to myelin sheath or axonal integrity) which would not necessary be reflected in volume differences (i.e., the size and density of white matter). Moreover, effects on white matter microstructure might occur earlier, and as such reflect early-stage effects, in comparison to effects on white matter volume that may manifest later and be indicative of more chronic effects. Together, these findings provide some support for the growing body of literature linking neonatal inflammation to changes in white matter development, extending these implications to the general population (Guma et al., 2019).

In contrast, other brain associations identified by Conole and colleagues in preterm infants (e.g., hippocampus (Conole et al., 2023)) were not observed in our study. This may suggest that the more severe inflammatory environment to which preterm infants may be exposed (as captured by the MPS-CRP) could result in more widespread neurodevelopmental alterations compared to those born at term (Gomez-Lopez et al., 2014). Of note, we tested interactions with gestational age (measured continuously) but did not find that it moderated associations between the MPS-CRP and brain outcomes. This could be due to the fact that although there was variability in gestational age within our sample, the vast majority of children were still born at term (or late pre-term), with less than 5 % born preterm (<37 weeks) and less than 1 % born extremely preterm (<28 weeks).

As with brain outcomes, we did not observe any associations between the MPS-CRP at birth and child psychiatric symptoms on average or over time within the general population. Interestingly, one previous study also in the general population did not observe direct associations between the MPS-CRP at birth and higher internalizing and externalizing symptoms during development (age 7–15 years), although indirect effects were identified through cognitive function and sustained inflammation (Barker et al., 2018). While these results may have been attributable to the large interval between the assessment of MPS-CRP and the behavioral outcomes in that study (i.e., with direct effects weakening over time), we assessed behavior repeatedly from early toddlerhood (age 1.5 years) to adolescence (age 14 years), but we did not find any evidence of stable or waning effects. Moreover, the absence of an association between MPS-CRP and behavioral outcomes in the child may stem from limitations related to the assumption that MPS-CRP at birth accurately reflects early life inflammatory status. Given the weak association with serum CRP at birth, it is challenging to determine whether MPS-CRP is effectively capturing inflammation or if the issue lies with the validity and limited variability of the CRP measurements at birth. In the future, it would be interesting to validate MPS-CRP at birth with other inflammatory biomarkers that have been validated in cord blood such as procalcitonin (Dongen et al., 2021). Additionally, it would be of interest to examine whether the MPS-CRP associates with other phenotypes linked to early inflammation (e.g., schizophrenia (Mongan et al., 2020)). Furthermore, the use of repeatedly assessed epigenetic data would help to clarify whether MPS-CRP may carry more information about neurobehavioral or psychiatric risk as children age (e.g., by also capturing relevant postnatal inflammatory exposures) and if this association over time is unidirectional or bidirectional.

### 4.4. Limitations

Our findings should be interpreted in light of several limitations. First, given the lack of variation in serum CRP in cord blood, our validation analyses should be considered with caution. This issue, however, is not specific to our study and reflects common challenges in reliably measuring serum CRP levels in neonates (Yadav et al., 2023), which explains the motivation to find alternative markers of neonatal inflammation, such as other serum measures or epigenetic proxies. Second, we were unable to examine prenatal predictors individually – opting instead for a cumulative score approach – due to the low prevalence of many exposures (e.g., pregnancy-related inflammatory conditions), which means we are unable to identify specific factors at specific timepoints that may associate with the MPS-CRP. Further, although we examined a wide range of prenatal factors and child genetic liability to elevated serum CRP levels, we did not have data on other exposures, such as placental conditions, which may explain variability in the MPS-CRP. Third, given the observational design of our study we cannot infer any causal relationships. Finally, the use of repeated MPS-CRP measures could in future help to clarify how inflammation and neurobehavioral outcomes relate to one another during development.

### 4.5. Conclusion

In conclusion, in this large-scale population-based cohort study, we provide novel insights into prenatal predictors and neurobehavioral outcomes of neonatal inflammation – measured with an epigenetic proxy of CRP (MPS-CRP). We demonstrated adequate predictive performance of MPS-CRP at birth for serum CRP at birth. However, since 94 % of the cord blood samples had undetectable CRP levels, the conclusions are limited, and further validation is necessary. We found that prenatal maternal lifestyle factors (e.g., smoking, BMI) – but not other factors including prenatal stress, prenatal infections, pregnancy-related inflammatory conditions, or the child’s genetic liability of elevated CRP levels – associated with the neonatal MPS-CRP within the general population. Moreover, in line with prior literature in animal models and preterm infants, we found suggestive evidence for a prospective association between elevated levels of MPS-CRP at birth and poorer white matter integrity from childhood to adolescence. Future research directions may involve following-up of these associations to examine if they persist or normalize during late adolescence. That said, our study found no evidence for a long-term association between MPS-CRP at birth and behavioral symptoms across development. While these findings may be reassuring, it will be important to extend analyses to later follow-up periods (e.g., late adolescence, adulthood) to examine potential associations with additional brain-based phenotypes (e.g., schizophrenia, neurological problems, cognitive function) linked to early inflammation.

Overall, our findings suggest that the MPS-CRP may indeed tag neonatal inflammation at birth, but that based on the predictors and outcomes examined it seems to capture limited information about inflammatory exposures and neurobehavioral risk within the general population.

## Supplementary Material

Supplementary material file 1

Supplementary material file 2

## Figures and Tables

**Table 1:** Demographic information participants.

	Aim 1 sample (n = 2,394)

**Maternal characteristics**	
Age mother at enrollment (mean, SD)	31.5 (4.3)
Pre-pregnancy BMI (mean, SD)	23.1 (3.8)
*Maternal education (n, %)*	
Low	74 (3.1)
Intermediate	830 (34.7)
High	1490 (62.2)
*Household income (n, %)*	
< €2200 per month	612 (25.6)
> €2200 per month	1782 (74.4)
*Smoking habits (n, %)*	
Never smoked during pregnancy	1798 (75.1)
Smoked until pregnancy was known	224 (9.4)
Continued smoking in pregnancy	372 (15.5)
**Child characteristics**	
Child’s sex, female (n, %)	1208 (50.5)
Gestational age at birth (weeks) (mean, SD)	40.1 (1.5)

**Table 2:** Linear regression between prenatal predictors and MPS-CRP (aim 1).

	Outcome	Standardized β-coefficient	Standard error	P-value

***MPS-CRP of Ligthart***	*Main model*			
	Prenatal stress score	−0.005	0.018	0.794
	Prenatal infection score	0.006	0.014	0.684
	Lifestyle pro-inflammatory factors score	0.033	0.015	0.027*
	Pregnancy related inflammatory clinical score	−0.009	0.014	0.540
	*Sensitivity analysis model (*i.e., *model without cell type proportions)*			
	Prenatal stress score	0.037	0.025	0.139
	Prenatal infection score	−0.012	0.020	0.542
	Lifestyle pro-inflammatory factors score	0.016	0.021	0.437
	Pregnancy related inflammatory clinical score	−0.099	0.02	<0.0001*

* p < 0.05.

Of note, because of the high correlation between the pregnancy related inflammatory clinical score and the medical inflammatory conditions score (r > 0.9), we omitted the medical inflammatory conditions score from the multivariate model.

**Table 3:** Linear mixed-effects model MPS-CRP and child brain development (aim 2).

	Outcome	Standardized β-coefficient	Standard error	P-value

***MPS-CRP of Ligthart***	*Main models*			
	Total brain volume	0.003	0.040	0.949
	Gray matter volume	0.009	0.040	0.815
	White matter volume	0.000	0.041	0.996
	Brain stem volume	−0.048	0.040	0.227
	Hippocampus volume	−0.024	0.044	0.588
	Amygdala volume	−0.022	0.044	0.615
	Lateral ventricles volume	−0.023	0.048	0.636
	Cerebellum volume	−0.085	0.041	0.039*
	Global mean diffusivity	−0.014	0.042	0.744
	Global fractional anisotropy	−0.025	0.046	0.592
	*Interaction modes (*i.e. *model with interaction time)l*			
	Total brain volume	−0.005	0.003	0.077
	Gray matter volume	−0.007	0.004	0.054
	White matter volume	−0.002	0.002	0.293
	Brain stem volume	−0.001	0.004	0.794
	Hippocampus volume	0.001	0.005	0.797
	Amygdala volume	0.016	0.006	0.010*
	Lateral ventricles volume	0.001	0.003	0.784
	Cerebellum volume	−0.007	0.003	0.036*
	Global mean diffusivity	0.004	0.006	0.531
	Global fractional anisotropy	−0.014	0.007	0.032*

* p < 0.05.

** p_fdr_ < 0.05.

**Table 4:** Linear mixed-effects model MPS-CRP and child psychiatric symptoms development (aim 3).

	Outcome	Standardized β-coefficient	Standard error	P-value

***MPS-CRP of Ligthart***	*Main models*			
	CBCL total behavioral symptoms	0.023	0.030	0.444
	CBCL internalizing symptoms	0.016	0.028	0.559
	CBCL externalizing symptoms	0.012	0.027	0.647
	*Interaction models (*i.e., *model with interaction time)*			
	CBCL total behavioral symptoms	−0.001	0.002	0.581
	CBCL internalizing symptoms	0.000	0.002	0.948
	CBCL externalizing symptoms	0.001	0.002	0.423

* p < 0.05.

** p_fdr_ < 0.05.

## Data Availability

The authors do not have permission to share data.
