# Supplementary material for: Mapping prenatal predictors and neurobehavioral outcomes of an epigenetic marker of neonatal inflammation – A longitudinal population-based study
Source: Brain Behav Immun. Author manuscript; Available in PMC 2025 Jan 31. (PMC11784988; doi:10.1016/j.bbi.2024.08.053)
Supplement: Supplementary material file 2 [file NIHMS2048131-supplement-Supplementary_material_file_2.docx]

**Mapping Prenatal Predictors and Neurobehavioral Outcomes of an Epigenetic Marker of Neonatal Inflammation – A Longitudinal Population-Based Study**

Anna Suleri^1,2^, Nicole Creasey^1,2,3^, Esther Walton^4^, Ryan Muetzel^1,5^, Janine F. Felix^2,6^, Liesbeth Duijts^7,8^, Veerle Bergink^9,10^, Charlotte Cecil^1,11,12^*

^1^Department of Child and Adolescent Psychiatry/Psychology, Erasmus MC, University Medical Center Rotterdam, Rotterdam, The Netherlands.

^2^The Generation R Study Group, Erasmus MC, University Medical Center Rotterdam, Rotterdam, the Netherlands.

^3^Department of Clinical, Educational & Health Psychology, Division of Psychology & Language sciences, Faculty of Brain Sciences, University College London, London, UK.

^4^Department of Psychology, University of Bath, Bath, United Kingdom.

^5^Department of Radiology and Nuclear Medicine, Erasmus MC, University Medical Center Rotterdam, Rotterdam, The Netherlands.

^6^Department of Pediatrics, Erasmus MC, University Medical Center Rotterdam, Rotterdam, The Netherlands.

^7^Department of Pediatrics, Division of Respiratory Medicine and Allergology, Erasmus MC, University Medical Center Rotterdam, Rotterdam, the Netherlands.

^8^Department of Neonatal and Pediatric Intensive Care, Division of Neonatology, Erasmus MC, University Medical Center Rotterdam, Rotterdam, the Netherlands.

^9^Department of Psychiatry, Icahn School of Medicine at Mount Sinai, New York, USA.

^10^Department of Psychiatry, Erasmus MC, University Medical Center Rotterdam, Rotterdam, The Netherlands.

^11^Department of Epidemiology, Erasmus MC, University Medical Center Rotterdam, Rotterdam, the Netherlands.

^12^Department of Biomedical Data Sciences, Molecular Epidemiology, Leiden University Medical Center, Leiden, The Netherlands.

*Corresponding author: [c.cecil@erasmusmc.nl](mailto:c.cecil@erasmusmc.nl)

**SUPPLEMENTARY INFORMATION**

**SUPPLEMENTARY TEXT**

1. Preregistration deviations

First, in the statistical analysis section of the preregistration we mentioned we would adjust for array and combine the 450K and EPIC samples. However, after additional checks after seeing the data, we decided to perform the analysis on the 450K and EPIC samples individually and then meta-analyze the results to minimize potential noise. Second, we added the PGS of CRP to the prenatal factors in aim 1 to better understand predictors of MPS-CRP. Third, because we did not have information on auto immune diseases, we instead used diabetes mellitus in the medical inflammatory conditions score. Fourth, in contrast to the preregistration we did not exclude mothers with missing data on prenatal infections, but we imputed the missing data using passive imputation to retain the highest possible sample. Fifth, because of the high multicollinearity between the pregnancy related inflammatory conditions score and the medical inflammatory conditions score and the subsequent violation of model assumptions in aim 1, we only included pregnancy related inflammatory conditions score in the multivariate model. Sixth, we excluded offspring sMRI and DTI data with poor quality data or incidental findings or braces. Seventh, we additionally adjusted for socioeconomic status as a covariate in aim 1. Eighth, in addition to examining prenatal factors associated with MPS-CRP at birth, we performed an additional sensitivity analysis for aim 3. This analysis explored whether MPS-CRP at birth is associated with inflammatory phenotypes in childhood, including asthma, allergy, eczema, and SDS-BMI. Finally, in contrast to what we specified in the preregistration, we did not perform a mediation analysis for 'gestational age at birth.' This decision was based on the weak evidence we found regarding direct effects between MPS-birth and neurobehavioral outcomes. Although there may still be an indirect effect, we did not observe any interaction between MPS-CRP at birth and gestational age in our models, making it unlikely that it is a mediator. Additionally, since less than 5% of the sample was born preterm, we likely lack the power to detect any mediation effects based on gestational age.

1. Processing and quality assessment DNA methylation data

DNA extracted (using the salting-out method) from blood samples taken at birth (cord blood). 500 ng DNA per sample underwent bisulfite conversion using the EZ-96 DNA Methylation kit (Shallow) (Zymo Research Corporation, Irvine, USA). Samples were plated onto 96-well plates in no specific order. For assessing DNA methylation in cord blood, we employed the Illumina Infinium® HumanMethylation450 and EPICv1 BeadChip technology. The preparation and normalization of DNA methylation data were executed following the CPACOR workflow (73) utilizing the R software package (The R Core Team, 2013). In detail, the idat files were read using the minfi package (74). Probes with a detection p-value above the background threshold (based on the sum of methylated and unmethylated intensity values) of 1E-16 or higher were marked as missing for each array. Following this step, the intensity values were stratified based on autosomal and non-autosomal probes and subsequently quantile normalized for each of the six probe type categories: type II red/green, type I methylated red/green, and type I unmethylated red/green. Beta values were computed as the ratio of the methylated intensity value to the sum of methylated, unmethylated, and an offset of 100 intensities. Arrays exhibiting technical issues, such as problems with bisulfite conversion, hybridization, or extension, were excluded from subsequent analyses. Arrays where the sex determination based on the chromosome X and Y probe intensities did not match the sex of the proband were also removed. Furthermore, only arrays with a sample call rate exceeding 95% and 96% for the 450k and EPICv1 arrays, respectively were taken forward for further processing.

1. Image processing and quality assessment sMRI and DTI scans

*Image processing*

sMRI: The FreeSurfer analysis suite (version 6.0.0) [http://surfer.nmr.mgh.harvard.edu/] was utilized to process the data at all timepoints. Briefly, the raw DICOM data was converted to MGZ files, followed by skull stripping, intensity normalization (correcting voxel intensities for B1 inhomogeneities), and voxel-based segmentation to distinguish gray matter, white matter, and cerebrospinal fluid.

DTI: Probabilistic fiber tractography was conducted on each child's native space diffusion data using the FSL plugin 'AutoPtx'. This plugin automatically identifies connectivity distributions for commonly reported fiber bundles. A nonlinear transformation from the FMRIB FA map was applied to generate connectivity distributions in each child’s native space. The number of successful seed-to-target attempts was used to normalize these connectivity distributions.

*Quality assurance*

To ensure scan accuracy, a trained evaluator visually examined the scans, focusing on the gray and white matter boundaries identified by FreeSurfer. Each scan was rated on a Likert scale as poor, questionable, or good, and low-quality scans were excluded from the analysis.

First imaging wave: Processed images were evaluated using a six-point Likert scale (0 = unusable to 5 = excellent). The evaluation criteria included cerebellar foliation, gray/white matter interface clarity, ringing artifacts, and subcortical structure segmentation (caudate and putamen). Excellent images had clear anterior and posterior cerebellar foliation, while poor images showed blurred interfaces and artifacts. Scans rated as poor or unusable were excluded.

Second imaging wave: Two independent raters assessed images using a three-point Likert scale (good, questionable, or poor). Consistently poor images were excluded. Disagreements were resolved by a third rater or expert consensus. A subset was also compared with automated quality assessment scores from a machine learning algorithm, which showed high sensitivity (83%) and moderate specificity (74%). Scans were included if both human and automated assessments rated them as good. Discrepant or unusable automated assessments were reviewed by an additional rater.

DTI: Both manual and automated quality control were applied. Manual assessment involved visual inspection of tensor calculation errors and tract reconstructions. Automated assessment used translation and rotation motion parameters from the eddy tool to exclude data from children with excessive motion.

**SUPPLEMENTARY FIGURES**


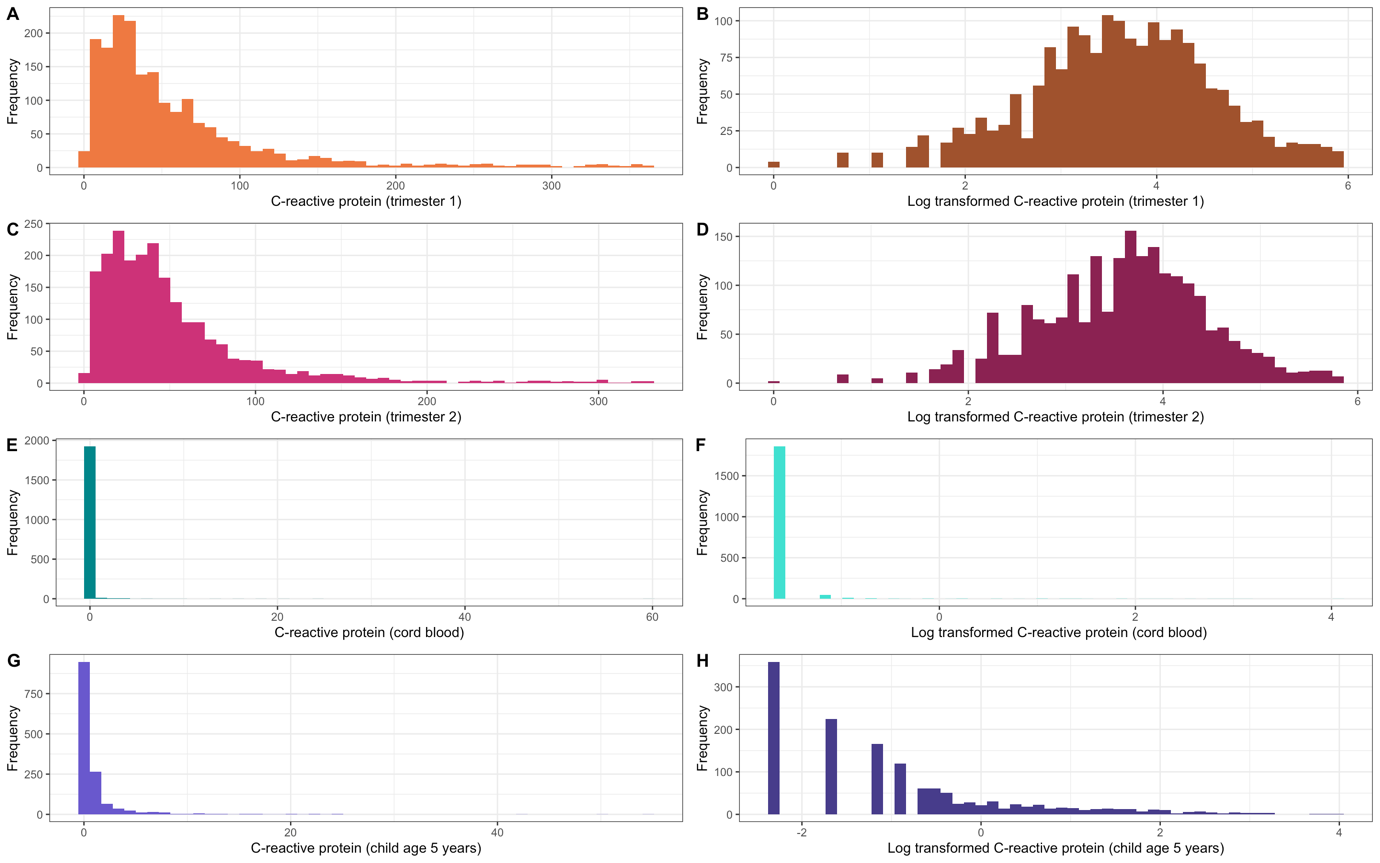


**Figure S1**. Distribution plots of serum C-reactive protein in trimester 1 and 2, cord blood and at child mean age 5 years. The untransformed (original scale, left column) and transformed (log scale, right column) distributions are shown. The median (1^st^ quartile – 3^rd^ quartile) for untransformed CRP in trimester 1 is 4.4 (2.3 – 7.8) mg/L, for CRP in trimester 2 is 3.2 (2.4 – 7.0) mg/L, for CRP in cord blood is 0.2 (0.2 – 0.2) mg/L and for CRP in the child at mean age 5 is 0.3 (0.1 – 0.9) mg/L.

**Figure S2**. Distribution plots prenatal scores.

**Figure S3**. Radar chart prenatal clinical scores. This chart shows the driving factors within each score.

**Figure S4**. Directed acyclic graph (DAG) of included covariates.


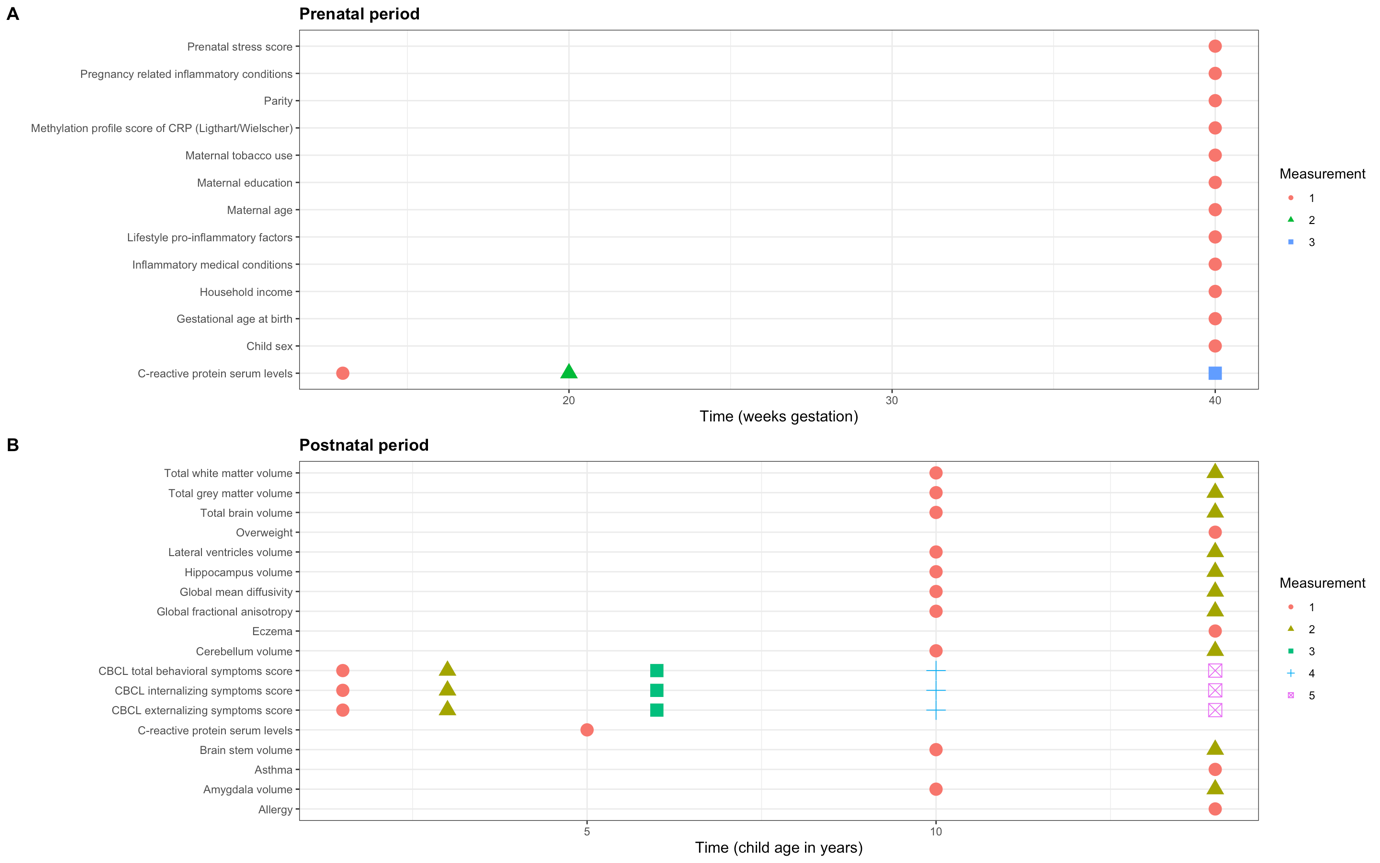


**Figure S5**. Data collection time point plot for each study variable. Plot S4A shows all the variables that were measured prenatally or at birth and plot S4B shows the variables that were measured postnatally.


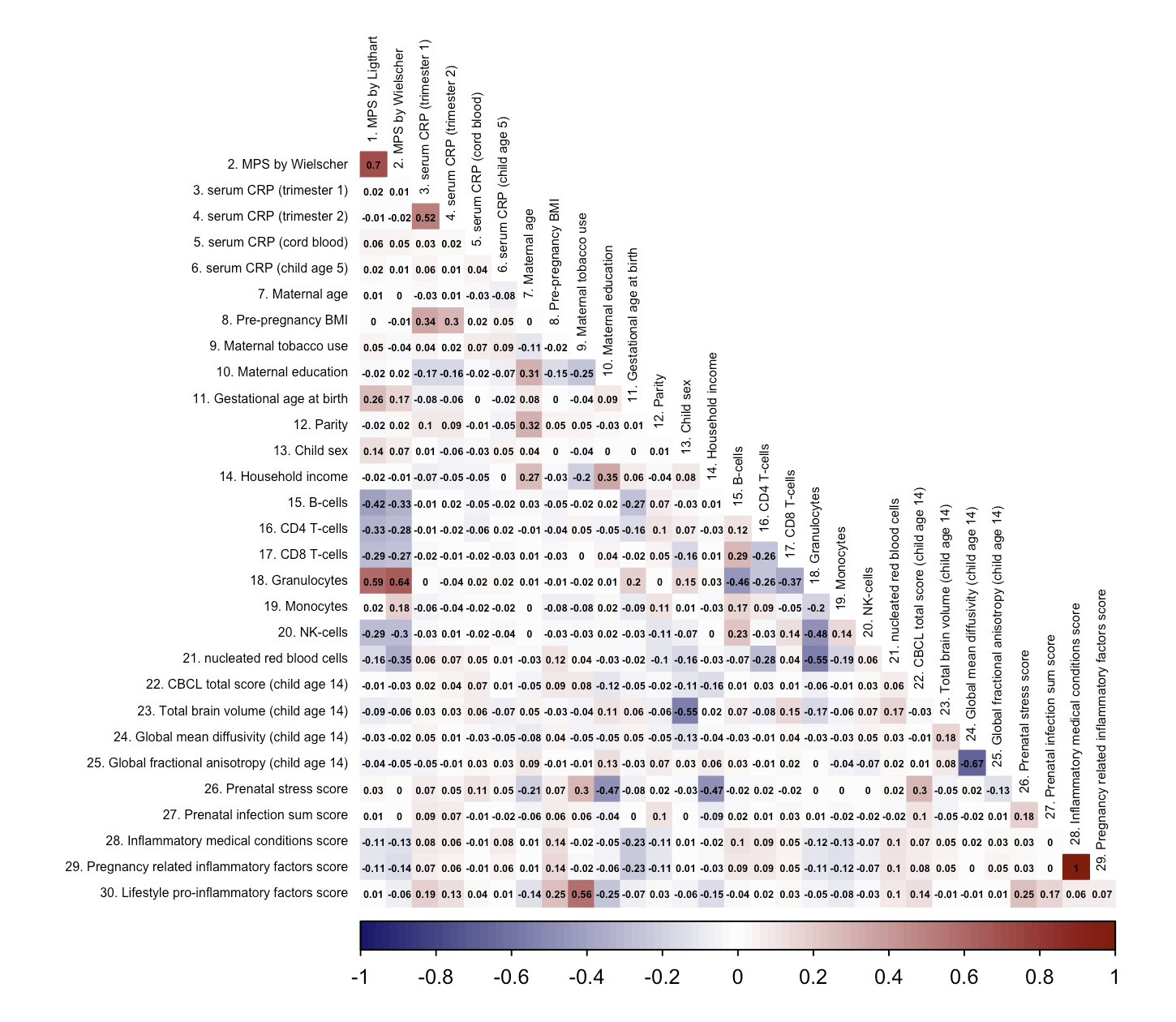


**Figure S6**. Correlation plot for the MPS-CRP of Ligthart (main MPS) and Wielscher (extended MPS), clinical inflammatory scores, cell types, and global brain and behavior outcomes. Of note, the MPS scores are residualized for batch effects and standardized.

**Figure S7**. Distribution plots of MPS-CRP with Ligthart (main MPS) and Wielscher (extended MPS) CpG sites based on 450K and EPIC assays.

**Figure S8**. Receiver operating characteristics curve with the area under the curve for the MPS-CRP of Ligthart (main MPS) and Wielscher (extended MPS) (similar with or without residualizing for batch effects) with the 450K and EPIC assays and serum C-reactive protein in cord blood (binary variable, cut-off<1mg/L).

**Figure S9**. Scatter plot for MPS-CRP scores versus (untransformed) serum CRP in cord blood. Of note, the MPS scores are residualized for batch effects and standardized.

**Figure S10**. Descriptive plot for aim 1, stratified by assay, showing the association prenatal predictors to the MPS-CRP of Ligthart (main MPS) and Wielscher (extended MPS). Of note, the MPS scores are residualized for batch effects. Alle scores (both of MPS and prenatal predictors are standardized).

**S11**. Scatter plot for PGS of CRP versus serum CRP.

**S12**. Assay specific plots for suggestive findings in aim 2. Ligthart is the main MPS and Wielscher is the extended MPS.

**S13**. Variance partition analysis for aim 1 (main & extended MPS). Ligthart is the main MPS and Wielscher is the extended MPS.

**S14**. Primary analyses results for each aim. A single asterisk indicates p<0.05 and a double asterisk indicates p_fdr_<0.05. Ligthart is the main MPS and Wielscher is the extended MPS.

**S15**. Suggestive findings aim 2. * Indicates p<0.05 and ** indicates p_fdr_<0.05. Ligthart is the main MPS and Wielscher is the extended MPS.

**SUPPLEMENTARY TABLES**

**Table S1**. Weights for MPS-CRP based on Ligthart EWAS (main MPS).

See Excel file.

**Table S2**. Univariate regression CpG sites Ligthart EWAS.

See Excel file.

**Table S3**. Weights for MPS-CRP based on Wielscher EWAS (extended MPS).

See Excel file.

**Table S4**. Univariate regression CpG sites Wielscher EWAS.

See Excel file.

**Table S5**. Linear regression between serum CRP and MPS-CRP.

|  | **Outcome** | **Standardized β-coefficient** | **Standard error** | **P-value** |
| --- | --- | --- | --- | --- |
| ***MPS-CRP of Ligthart (main)*** | *Univariate model* | | | |
|  | Serum CRP in trimester 1 | 0.017 | 0.023 | 0.477 |
|  | Serum CRP in trimester 2 | -0.024 | 0.024 | 0.256 |
|  | Serum CRP in cord blood | 0.046 | 0.023 | 0.049* |
|  | Serum CRP at child age 5 | 0.028 | 0.027 | 0.294 |
|  | *Batch effects adjusted model* | | | |
|  | Serum CRP in trimester 1 | 0.020 | 0.026 | 0.448 |
|  | Serum CRP in trimester 2 | -0.015 | 0.024 | 0.535 |
|  | Serum CRP in cord blood | 0.071 | 0.026 | 0.007** |
|  | Serum CRP at child age 5 | 0.022 | 0.03 | 0.478 |
|  | *Batch effects and cell type proportions adjusted model* | | | |
|  | Serum CRP in trimester 1 | 0.042 | 0.038 | 0.269 |
|  | Serum CRP in trimester 2 | 0.008 | 0.035 | 0.821 |
|  | Serum CRP in cord blood | 0.072 | 0.038 | 0.057 |
|  | Serum CRP at child age 5 | 0.027 | 0.045 | 0.546 |
| ***MPS-CRP of Wielscher (extended)*** | *Univariate model* | | | |
|  | Serum CRP in trimester 1 | 0.010 | 0.023 | 0.683 |
|  | Serum CRP in trimester 2 | -0.023 | 0.024 | 0.278 |
|  | Serum CRP in cord blood | 0.033 | 0.023 | 0.144 |
|  | Serum CRP at child age 5 | 0.024 | 0.026 | 0.363 |
|  | *Batch effects adjusted model* | | | |
|  | Serum CRP in trimester 1 | 0.015 | 0.028 | 0.592 |
|  | Serum CRP in trimester 2 | -0.022 | 0.026 | 0.383 |
|  | Serum CRP in cord blood | 0.052 | 0.027 | 0.054 |
|  | Serum CRP at child age 5 | 0.008 | 0.032 | 0.804 |
|  | *Batch effects and cell type proportions adjusted model* | | | |
|  | Serum CRP in trimester 1 | 0.074 | 0.046 | 0.108 |
|  | Serum CRP in trimester 2 | 0.020 | 0.043 | 0.643 |
|  | Serum CRP in cord blood | 0.060 | 0.045 | 0.179 |
|  | Serum CRP at child age 5 | -0.003 | 0.054 | 0.959 |
| *p<0.05  **p_fdr_<0.05 | | | | |

**Table S6**. PGS of CRP validation.

| **Outcome** | **Spearman correlation** |
| --- | --- |
| Serum CRP in trimester 1 | 0.221* |
| Serum CRP in trimester 2 | 0.209* |
| Serum CRP in cord blood | 0.044 |
| Serum CRP in child age 5 | 0.233* |
| *p<0.05 | |

**Table S7**. Association between PGS of CRP and MPS-CRP (aim 1).

|  | **Predictor** | **Standardized β-coefficient** | **Standard error** | **P-value** |
| --- | --- | --- | --- | --- |
| ***MPS-CRP of Ligthart (main)*** | *Main model* | | | |
|  | PGS of CRP | 0.012 | 0.013 | 0.358 |
|  | *Sensitivity analysis model (i.e., model without cell type proportions)* | | | |
|  | PGS of CRP | 0.023 | 0.023 | 0.328 |
| ***MPS-CRP of Wielscher (extended)*** | *Main model* | | | |
|  | PGS of CRP | -0.0027 | 0.011 | 0.328 |
|  | *Sensitivity analysis model (i.e., model without cell type proportions)* | | | |
|  | PGS of CRP | 0.006 | 0.021 | 0.775 |
| *p<0.05  **p_fdr_<0.05 | | | | |

**Table S8**. Linear mixed-effects model MPS of CRP and child brain development (aim 2).

|  | **Outcome** | | **Standardized β-coefficient** | | **Standard error** | **P-value** | |
| --- | --- | --- | --- | --- | --- | --- | --- |
| ***MPS-CRP of Ligthart (main)*** | *Main models + intercranial volume* | | | | | | |
|  | Gray matter volume | 0.035 | | 0.021 | | | 0.101 |
|  | White matter volume | 0.025 | | 0.023 | | | 0.269 |
|  | Brain stem volume | -0.032 | | 0.032 | | | 0.323 |
|  | Hippocampus volume | -0.01 | | 0.037 | | | 0.781 |
|  | Amygdala volume | -0.007 | | 0.036 | | | 0.841 |
|  | Lateral ventricles volume | -0.013 | | 0.044 | | | 0.773 |
|  | Cerebellum volume | -0.072 | | 0.037 | | | 0.048* |
|  | *Moderation models: gestational age at birth* | | | | | | |
|  | Total brain volume | | -0.001 | | 0.003 | 0.733 | |
|  | Gray matter volume | | 0.000 | | 0.004 | 0.968 | |
|  | White matter volume | | -0.002 | | 0.003 | 0.572 | |
|  | Brain stem volume | | 0.001 | | 0.004 | 0.811 | |
|  | Hippocampus volume | | 0.005 | | 0.005 | 0.342 | |
|  | Amygdala volume | | 0.004 | | 0.006 | 0.568 | |
|  | Lateral ventricles volume | | 0.003 | | 0.003 | 0.354 | |
|  | Cerebellum volume | | 0.001 | | 0.004 | 0.830 | |
|  | Global mean diffusivity | | -0.001 | | 0.006 | 0.810 | |
|  | Global fractional anisotropy | | 0.004 | | 0.007 | 0.567 | |
|  | *Sensitivity analysis models (i.e., model without cell type proportions)* | | | | | | |
|  | Total brain volume | | -0.017 | | 0.028 | 0.530 | |
|  | Gray matter volume | | -0.007 | | 0.027 | 0.790 | |
|  | White matter volume | | -0.025 | | 0.028 | 0.379 | |
|  | Brain stem volume | | -0.016 | | 0.027 | 0.549 | |
|  | Hippocampus volume | | -0.027 | | 0.030 | 0.369 | |
|  | Amygdala volume | | -0.026 | | 0.030 | 0.380 | |
|  | Lateral ventricles volume | | -0.02 | | 0.033 | 0.536 | |
|  | Cerebellum volume | | -0.014 | | 0.028 | 0.620 | |
|  | Global mean diffusivity | | -0.023 | | 0.029 | 0.424 | |
|  | Global fractional anisotropy | | -0.007 | | 0.031 | 0.819 | |
| ***MPS-CRP of Wielscher (extended)*** | *Main models + intercranial volume* | | | | | | |
|  | Gray matter volume | 0.023 | | 0.027 | | | 0.398 |
|  | White matter volume | 0.019 | | 0.029 | | | 0.511 |
|  | Brain stem volume | 0.035 | | 0.041 | | | 0.391 |
|  | Hippocampus volume | 0.012 | | 0.047 | | | 0.797 |
|  | Amygdala volume | -0.058 | | 0.046 | | | 0.208 |
|  | Lateral ventricles volume | -0.061 | | 0.056 | | | 0.271 |
|  | Cerebellum volume | 0.014 | | 0.047 | | | 0.772 |
|  | *Moderation models: gestational age at birth* | | | | | | |
|  | Total brain volume | | -0.004 | | 0.003 | 0.141 | |
|  | Gray matter volume | | -0.004 | | 0.004 | 0.285 | |
|  | White matter volume | | -0.004 | | 0.002 | 0.124 | |
|  | Brain stem volume | | 0.000 | | 0.004 | 0.903 | |
|  | Hippocampus volume | | 0.000 | | 0.005 | 0.996 | |
|  | Amygdala volume | | 0.002 | | 0.006 | 0.714 | |
|  | Lateral ventricles volume | | 0.001 | | 0.003 | 0.828 | |
|  | Cerebellum volume | | -0.001 | | 0.003 | 0.701 | |
|  | Global mean diffusivity | | -0.003 | | 0.006 | 0.629 | |
|  | Global fractional anisotropy | | 0.007 | | 0.006 | 0.301 | |
|  | *Sensitivity analysis models (i.e., model without cell type proportions)* | | | | | | |
|  | Total brain volume | | -0.004 | | 0.030 | 0.883 | |
|  | Gray matter volume | | 0.001 | | 0.030 | 0.978 | |
|  | White matter volume | | -0.011 | | 0.031 | 0.716 | |
|  | Brain stem volume | | 0.021 | | 0.030 | 0.493 | |
|  | Hippocampus volume | | -0.007 | | 0.033 | 0.839 | |
|  | Amygdala volume | | -0.032 | | 0.032 | 0.330 | |
|  | Lateral ventricles volume | | -0.047 | | 0.036 | 0.194 | |
|  | Cerebellum volume | | 0.034 | | 0.030 | 0.277 | |
|  | Global mean diffusivity | | -0.011 | | 0.031 | 0.735 | |
|  | Global fractional anisotropy | | -0.031 | | 0.034 | 0.366 | |
| *p<0.05  **p_fdr_<0.05 | | | | | | | |

**Table S9**. Linear mixed-effects model MPS of CRP and child psychiatric symptoms (aim 3).

|  | **Outcome** | **Standardized β-coefficient** | **Standard error** | **P-value** |
| --- | --- | --- | --- | --- |
| ***MPS-CRP of Ligthart (main)*** | *Moderation models: gestational age at birth* | | | |
|  | CBCL total behavioral symptoms | -0.003 | 0.002 | 0.038* |
|  | CBCL internalizing symptoms | -0.003 | 0.002 | 0.116 |
|  | CBCL externalizing symptoms | -0.003 | 0.002 | 0.056 |
|  | *Sensitivity analysis models (i.e., model without cell type proportions)* | | | |
|  | CBCL total behavioral symptoms | 0.017 | 0.021 | 0.420 |
|  | CBCL internalizing symptoms | 0.021 | 0.020 | 0.283 |
|  | CBCL externalizing symptoms | 0.007 | 0.019 | 0.711 |
| ***MPS-CRP of Wielscher (extended)*** | *Moderation model: gestational age at birth* | | | |
|  | CBCL total behavioral symptoms | -0.003 | 0.002 | 0.129 |
|  | CBCL internalizing symptoms | -0.002 | 0.002 | 0.282 |
|  | CBCL externalizing symptoms | -0.003 | 0.002 | 0.125 |
|  | *Sensitivity analysis models (i.e., model without cell type proportions)* | | | |
|  | CBCL total behavioral symptoms | -0.011 | 0.022 | 0.608 |
|  | CBCL internalizing symptoms | 0.012 | 0.021 | 0.571 |
|  | CBCL externalizing symptoms | -0.019 | 0.02 | 0.339 |
| *p<0.05  **p_fdr_<0.05 | | | | |

**Table S10**. Regression between MPS-CRP at birth and inflammatory outcomes in childhood.

|  | **Childhood outcome (mean age 14 years)** | **Effect estimate** | **Standard error** | **P-value** |
| --- | --- | --- | --- | --- |
| ***MPS-CRP of Ligthart (main)*** | Asthma (OR) | 1.141 | 1.155 | 0.359 |
|  | Allergy (OR) | 1.147 | 1.094 | 0.125 |
|  | Eczema (OR) | 1.136 | 1.139 | 0.328 |
|  | SDS-BMI (standardized β-coefficients) | -0.060 | 0.041 | 0.145 |
| ***MPS-CRP of Wielscher (extended)*** | Asthma (OR) | 0.900 | 0.173 | 0.544 |
|  | Allergy (OR) | 1.113 | 1.269 | 0.027* |
|  | Eczema (OR) | 1.055 | 1.167 | 0.731 |
|  | SDS-BMI (standardized β-coefficients) | -0.026 | 0.049 | 0.601 |
| OR = odds ratio  *p<0.05  **p_fdr_<0.05 | | | | |

**Table S11**. Linear regression between prenatal predictors and MPS-CRP (aim 1).

|  | **Outcome** | **Standardized β-coefficient** | **Standard error** | **P-value** |
| --- | --- | --- | --- | --- |
| ***MPS-CRP of Wielscher (extended)*** | *Main model* | | | |
|  | Prenatal stress score | -0.006 | 0.014 | 0.654 |
|  | Prenatal infection score | 0.015 | 0.012 | 0.195 |
|  | Lifestyle pro-inflammatory factors score | -0.001 | 0.012 | 0.965 |
|  | Pregnancy related inflammatory clinical score | -0.008 | 0.012 | 0.491 |
|  | *Sensitivity analysis model (i.e., model without cell type proportions)* | | | |
|  | Prenatal stress score | 0.029 | 0.023 | 0.214 |
|  | Prenatal infection score | 0.003 | 0.019 | 0.885 |
|  | Lifestyle pro-inflammatory factors score | -0.033 | 0.019 | 0.092 |
|  | Pregnancy related inflammatory clinical score | -0.108 | 0.018 | <0.0001** |
| *p<0.05  **p_fdr_<0.05  Of note, because of the high correlation between the pregnancy related inflammatory clinical score and the medical inflammatory conditions score (r>0.9), we omitted the medical inflammatory conditions score from the multivariate model. | | | | |

**Table S12**. Linear mixed-effects model MPS-CRP and child brain development (aim 2).

|  | **Outcome** | **Standardized β-coefficient** | **Standard error** | **P-value** |
| --- | --- | --- | --- | --- |
| ***MPS-CRP of Wielscher (extended)*** | *Main models* | | | |
|  | Total brain volume | 0.064 | 0.051 | 0.208 |
|  | Gray matter volume | 0.062 | 0.050 | 0.215 |
|  | White matter volume | 0.057 | 0.052 | 0.277 |
|  | Brain stem volume | 0.061 | 0.051 | 0.224 |
|  | Hippocampus volume | 0.039 | 0.055 | 0.475 |
|  | Amygdala volume | -0.028 | 0.055 | 0.611 |
|  | Lateral ventricles volume | -0.046 | 0.06 | 0.444 |
|  | Cerebellum volume | 0.034 | 0.052 | 0.512 |
|  | Global mean diffusivity | 0.040 | 0.053 | 0.449 |
|  | Global fractional anisotropy | -0.081 | 0.058 | 0.164 |
|  | *Interaction models (i.e., model with interaction time)* | | | |
|  | Total brain volume | -0.004 | 0.003 | 0.134 |
|  | Gray matter volume | -0.005 | 0.004 | 0.184 |
|  | White matter volume | -0.004 | 0.002 | 0.108 |
|  | Brain stem volume | -0.001 | 0.004 | 0.886 |
|  | Hippocampus volume | 0.002 | 0.004 | 0.665 |
|  | Amygdala volume | 0.012 | 0.006 | 0.044* |
|  | Lateral ventricles volume | 0.000 | 0.003 | 0.967 |
|  | Cerebellum volume | -0.005 | 0.003 | 0.087 |
|  | Global mean diffusivity | 0.001 | 0.006 | 0.807 |
|  | Global fractional anisotropy | -0.018 | 0.006 | 0.005** |
| *p<0.05  **p_fdr_<0.05 | | | | |

**Table S13**. Linear mixed-effects model MPS-CRP and child psychiatric symptoms development (aim 3).

|  | **Outcome** | **Standardized β-coefficient** | **Standard error** | **P-value** |
| --- | --- | --- | --- | --- |
| ***MPS-CRP of Wielscher (extended)*** | *Main models* | | | |
|  | CBCL total behavioral symptoms | -0.011 | 0.037 | 0.757 |
|  | CBCL internalizing symptoms | 0.013 | 0.035 | 0.713 |
|  | CBCL externalizing symptoms | -0.025 | 0.033 | 0.453 |
|  | *Interaction models (i.e., model with interaction time)* | | | |
|  | CBCL total behavioral symptoms | 0.001 | 0.002 | 0.572 |
|  | CBCL internalizing symptoms | 0.002 | 0.002 | 0.351 |
|  | CBCL externalizing symptoms | 0.003 | 0.002 | 0.074 |
| *p<0.05  **p_fdr_<0.05 | | | | |
